# Religious fasting and its impacts on individual, public, and planetary health: Fasting as a “religious health asset” for a healthier, more equitable, and sustainable society

**DOI:** 10.3389/fnut.2022.1036496

**Published:** 2022-11-24

**Authors:** Khaled Trabelsi, Achraf Ammar, Mohamed Ali Boujelbane, Luca Puce, Sergio Garbarino, Egeria Scoditti, Omar Boukhris, Saber Khanfir, Cain C. T. Clark, Jordan M. Glenn, Omar A. Alhaj, Haitham Jahrami, Hamdi Chtourou, Nicola Luigi Bragazzi

**Affiliations:** ^1^Research Laboratory: Education, Motricity, Sport and Health, Sfax, Tunisia; ^2^Higher Institute of Sport and Physical Education of Sfax, University of Sfax, Sfax, Tunisia; ^3^Department of Training and Movement Science, Institute of Sport Science, Johannes Gutenberg-University Mainz, Mainz, Germany; ^4^UFR SESS-STAPS, Paris-East Créteil University, LIRTES (EA 7313), Créteil, France; ^5^Department of Neuroscience, Rehabilitation, Ophthalmology, Genetics, Maternal and Child Health (DINOGMI), University of Genoa, Genoa, Italy; ^6^National Research Council, Institute of Clinical Physiology, Lecce, Italy; ^7^Sport and Exercise Science, School of Allied Health, Human Services and Sport, La Trobe University, Melbourne, VIC, Australia; ^8^Faculty of Medicine of Tunis, University of Tunis El Manar, Tunis, Tunisia; ^9^Centre for Intelligent Healthcare, Coventry University, Coventry, United Kingdom; ^10^Department of Health, Exercise Science Research Center Human Performance and Recreation, University of Arkansas, Fayetteville, AR, United States; ^11^Department of Nutrition, Faculty of Pharmacy and Medical Sciences, University of Petra, Amman, Jordan; ^12^Department of Psychiatry, Ministry of Health, Manama, Bahrain; ^13^Department of Psychiatry, College of Medicine and Medical Sciences, Arabian Gulf University, Manama, Bahrain; ^14^Laboratory for Industrial and Applied Mathematics, Department of Mathematics and Statistics, York University, Toronto, ON, Canada

**Keywords:** faith-based fasting, sustainability, nutrition, lifestyle, global health, public health

## Abstract

Religious fasting is practiced by people of all faiths, including Christianity, Islam, Buddhism, Jainism, as well as Hinduism, Judaism, and Taoism. Individual/clinical, public, global, and planetary health has traditionally been studied as separate entities. Nevertheless, religious fasting, in conjunction with other religious health assets, can provide several opportunities, ranging from the individual to the population, environmental, and planetary levels, by facilitating and supporting societal transformations and changes, such as the adoption of healthier, more equitable, and sustainable lifestyles, therein preserving the Earth's systems and addressing major interconnected, cascading, and compound challenges. In this review, we will summarize the most recent evidence on the effects of religious fasting, particularly Orthodox and Ramadan Islamic fasting, on human and public health. Further, we will explore the potential effects of religious fasting on tackling current environmental issues, with a special focus on nutrition/food restriction and planetary health. Finally, specific recommendations, particularly around dietary intake during the fasting rituals, will be provided to ensure a sustainable healthy planet.

## Introduction

Human life expectancy is substantially higher today as opposed to 100 years ago, mainly attributed to advances in both preventive and clinical medicine and public health policies (1). However, increased longevity is associated with a growth in non-communicable and disabling diseases of old age such as neurodegenerative diseases, cardiovascular disease, and cancer. Furthermore, the western diet and lifestyle, characterized by unhealthy diet and sedentariness, has engendered many, so-called diseases of civilization, including obesity-associated metabolic syndrome, coronary heart disease, hypertension, stroke, type 2 diabetes, chronic liver disease, autoimmune disease, epithelial cell cancers, and osteoporosis, which are scarce or non-existent in hunter-gatherers and other non-westernized populations (2). As a consequence of civilization, life expectancy is expected, for the first time, to fall (3, 4). Moreover, the current western dietary pattern, high in animal-based and low in plant-based foods, is harmful; not only to personal health (5, 6), but also to public, global, and planetary health (7, 8). In this context, previous studies show meal consumption is linked to premature mortality and type 2 diabetes (9–11). Moreover, meat has been recognized as the food with the highest influence on greenhouse gas (GHG) emissions and land usage (12). In this context, producing 1,000 kcal of lamb or beef creates 14 and 10 kg of GHG emissions, respectively, compared to 1 and 3 kg for lentils or tofu (13). Additionally, meat production is deleterious for aquifers (14); one serving of beef or pork requires 1211 and 469 L of water, respectively, but one serving of dry beans, tofu, or tomatoes requires 220, 57, and 30 L, respectively (13). Several studies have found that reducing meat consumption can reduce GHG emissions, as well as land, water, and energy use, while concomitantly improving health outcomes (12). Therefore, an extension of a healthy life, while tackling transnational environmental challenges, is of utmost importance.

Religious fasting, or faith-based fasting, is predominantly practiced to satisfy prescribed religious requirements and is defined as a nutritional model characterized by a variance in the degree of caloric restriction and abstinence from specific foods (15). Interestingly, religious fasting could help to improve individual health as well as the community, and planet. Fasting rituals are followed by billions of individuals worldwide and their effects may differ from one religious community to another (16). It should be acknowledged that 83% of the world's population self-identified as adhering to a religion in 2010, and this number is projected to rise to 87% by 2050 (17–19). In addition, Christians are the world's largest religious community, with Islam coming in second and being the fastest-growing faith (20). Skirbekk et al. (19) reported that countries with a greater proportion of religiously affiliated people are facing more environmental risks and are less prepared for those risks. Therefore, targeting the largest religious groups (i.e., Christians, Muslims) *via* specific recommendations may contribute to a “*healthy planet, supporting healthy people*.”

Using a comprehensive search, we will summarize in this review the latest evidence on the effects of religious fasting, particularly Orthodox and Ramadan Islamic fasting, on human health. Additionally, the potential effects of religious fasting on public and planetary health will be discussed and specific recommendations will be provided.

## Methods

A two-step search process was conducted to identify (i) systematic reviews, with/or without meta-analyses, or scoping reviews and (ii) experimental studies evaluating the effects of any type of religious fasting on health outcomes in apparently healthy individuals (not physically active individuals and athletes) and patients.

In the first step, to identify systematic reviews, with/or without meta-analyses, or scoping reviews, a systematic literature search was conducted in five databases (PubMed, Web of Science, Cochrane, EBSCOhost, Scielo) on August 10th 2022. An additional search on Google Scholar was conducted on August 15th 2022. The following terms were used (see Table S1): “Religious fasting,” “faith-based fasting,” “human,” “health,” “scoping review,” “systematic review,” and “meta-analysis.” Appropriate Boolean operators were used to join the various keywords. Field tags, wild-card options (i.e., truncated words), and medical subject headings (MeSH) terms were also incorporated where appropriate. In the search strategy, no language or date limits were applied. The full search strategy for all databases is presented in Table S1. References of included papers were manually verified for a comprehensive search for relevant reviews. Personal files were also searched.

In the second step, experimental studies were searched on August 10th 2022 in Google Scholar using the following terms: “Religious fasting,” “faith-based fasting,” “human” and “health.” To identify experimental studies published after the date of the last search of the most recent comprehensive review, date limits were applied.

## Results

**Figure 1:**
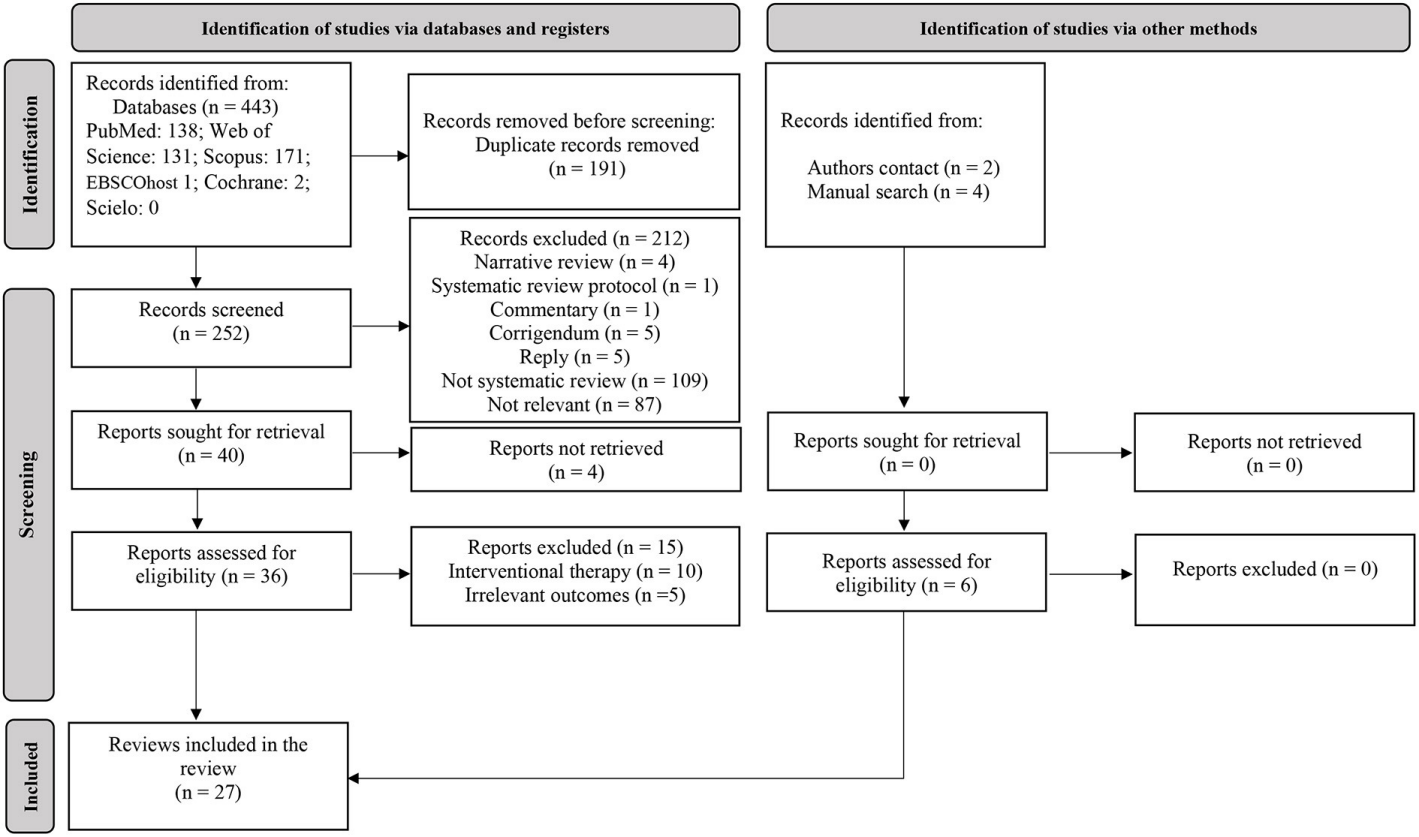
Flow diagram of search.

For the first-step, the predefined search strategies yielded a preliminary pool of 443 possible papers. A total of 191 duplicates were removed. Then, 252 published papers were screened by titles and abstracts for eligibility, of which 36 published studies met the inclusion criteria. After a careful review of the 36 full texts, 27 reviews (systematic review with or without a meta-analysis) were included (Figure 1). Of the 27 included reviews, only one systematic review (21) and a scoping review (22) evaluated the effects of Orthodox fasting on health/nutritional outcomes. The remainder reviews (*n* = 25) focused on the effects of Ramadan fasting on anthropometric and metabolic markers (23–25), glucometabolic parameters (26–29), salivary flow rate, inflammatory and metabolic variables (30), immunity, inflammatory markers and infectious events (31–33), blood pressure and cardiovascular events (34–36), liver function (37), renal function and chronic kidney diseases (38, 39), body mass (i.e., body weight) and body composition (40–42), intestinal microbiome changes (43), hormones regulating appetite and satiety (44), psychiatric parameters (45), sleep quality (46) and pregnancy outcomes (47). The characteristics and the main outcomes of the included reviews are presented in Table S2.

For the second-step, 53 research articles evaluating the effect of Ramadan fasting on anthropometric parameters (*n* = 10), hydration status (*n* = 11), renal function (*n* = 12), metabolic health (*n* = 9), liver function (*n* = 4), markers of inflammation, immunity and oxidative stress (*n* = 2), the gut microbiome (*n* = 3), and sleep (*n* = 2) were included. Other empirical studies evaluating the effects of Orthodox fasting (*n* = 13), Daniel (*n* = 3) and Buddhist (*n* = 2) fasting were included.

## Religious fasting, nutrition and individual health

In many religions, fasting is a fundamental practice with both spiritual and physical advantages (48). The health implications of religious fasting have been the topic of several scientific investigations, with the majority of studies conducted in the previous three decades. However, these implications have typically been studied at the individual level, with few studies exploring other domains across wellbeing from a transdisciplinary perspective. Besides the individual level of wellbeing (subjective hedonic and psychosocial wellbeing, “a balanced mind and a healthy body,” and spiritual wellbeing), fasting impacts the community's well-being (“social or collective wellbeing,” social capital, cohesion, connectedness, and “social identity” – a sense of belonging to a community), and the environmental and planetary wellbeing (“connection with nature,” nature-based mindfulness, “environmental and planetary wellbeing,” and nurturing environments). Over time, and throughout history, several religious actors and institutions, including trusted religious leaders, faith-based organizations, and faith communities, at all levels and of any religious creed/belief, have played a major role in the delivery of healthcare services and provisions and have contributed to shaping health emergency preparedness and response, as well as to mobilizing community-led action and catalyzing community partnership.

The effects of the religious Islamic fasting of Ramadan, the Greek Orthodox Christianity fasting, and the Biblical-based Daniel Fast, on subjects' dietary intake and health-related outcomes, will be outlined. Other forms of religious fasting (i.e., Buddhism, Daniel fast, Judaism) are only briefly overviewed in this review given the paucity of health-related data available.

### Islamic Ramadan fasting

During Ramadan fasting, one of the five pillars of the Islamic religion, more than 1.8 billion healthy pubescent Muslims worldwide must abstain from eating, drinking, and other specific behaviors (e.g., smoking, sexual intercourses, and all sensory pleasures) from dawn to sunset, during a period that lasts 29 or 30 days (lunar month) (15, 49). The daily fasting time length depends on the geographical location (latitude) and season. For example, in the north of the UK, daylight lasts <6 h in winter and about 19 h in midsummer (50).

During this month, water and food consumption are exclusively nocturnal and the typical dietary practice during Ramadan fasting consists of consuming one substantial meal after sunset and one lighter meal before dawn (51); however, an additional meal is sometimes consumed before sleeping (52). Interestingly, Ramadan fasting is similar to Alternate-Day Fasting in that the 12-h fast is followed by a 12-h feast. However, Ramadan fasting varies from Alternate-Day Fasting in that drinking water is not permitted throughout the 12-h fast (51, 53).

#### Food choices/preferences

Globally, there is large variability in dietary pattern between cultures in countries where individuals are observing Ramadan. Therefore, it is not surprising to observe different food preferences, specific to each culture and/or country. For example, Pirsaheb et al. (54) found, in a study of 160 Iranian subjects, there was a higher consumption of vegetables and fruits during Ramadan, whereas the consumption of dairy products, meat, and cereals decreased significantly. Contrarywise, in a study of 366 Ghanaian adolescents, it was found that the consumption of milk and vitamin A-rich fruits increased during Ramadan, while there was a lower consumption of dark leafy vegetables, legumes, and nuts (55). The authors also found that meat (beef, mutton, and chevon) consumption patterns remained relatively stable compared to before the month of Ramadan (~3 days/week), while poultry (chicken, guinea fowl, turkey) intake decreased marginally during Ramadan (55).

During a year-round study (56), including the month of Ramadan, Lebanese adults (*n* = 62), completed multiple (9–12, 14) 24-h dietary recalls. Dietary intake was estimated using food groups as well as energy, macro, and micronutrient consumption. The authors found that the intakes of cereals, cereal-based products, pasta, eggs, seeds and nuts, milk and dairy, and oils and fats were lower, while dried fruit, vegetables, cakes, pastries, and Arabic sweets, and sugar-sweetened-beverages intakes were higher during Ramadan as compared to the remainder of the year (56). In addition, the intake of poultry, meat, fish and seafood remained unchanged during vs. the remainder of the year (56). Nashvak et al. (57) evaluated the consumption of food groups before and during Ramadan in 160 healthy men from Iran. The authors found that, compared to before Ramadan, the consumption of bread and cereals, dairy products, meat, and vegetables decreased, while the consumption of fruit, fat, and oil increased during Ramadan.

#### Anthropometric parameters

During the month of Ramadan, food and fluid intake becomes less frequent and, therefore, fluctuations in some anthropometric variables are to be expected.

The first published meta-analysis evaluating the effect of Ramadan observance on body mass was by Kul et al. (23). The authors found that Ramadan fasting decreases body mass (small effect). In addition, the subgroup analysis indicated that the decrease in body mass was sex-specific as it was observed in men but not in women.

The aforementioned meta-analysis (23) was updated by Sadeghirad et al. (40); in the updated version, the authors showed Ramadan fasting elicited a statistically significant decrease in body mass (−1.24 kg by the end of Ramadan). Again, the decrease in body mass was significant in both sexes (−1.51 kg in men and −0.92 kg in women). However, the body mass loss during Ramadan was continent-specific, as it was greater among Asian populations compared with Africans and Europeans. Furthermore, the body mass loss observed at follow-up (2–6 weeks after Ramadan) was less pronounced, albeit body mass was still statistically significantly lower compared to before Ramadan (−0.72 kg).

The first systematic review with meta-analysis evaluating the effects of Ramadan fasting on body composition was conducted by Fernando et al. (41). The authors found that, from before to the end of Ramadan, there was a significant reduction in fat percentage in overweight or obese individuals, but not in those of normal weight. Similarly, Ramadan fasting yielded a significant loss of fat-free mass, but was about 30% less than the loss of absolute fat mass. After the end of Ramadan (2–5 weeks), there was a return toward, or to, pre-Ramadan measurements in body mass and body composition.

In another systematic review and meta-analysis, Jahrami et al. (42) concluded Ramadan fasting yielded a significant, but small reduction in body mass; this effect size translates into a difference in means of −1.022 kg. The regression analyses, used to explain sources of heterogeneity in the results, revealed that the decrease in body mass was directly associated with fasting time and correlated with country and season.

Regarding recent empirical studies, Agagündüz et al. (58) found, in 27 healthy Turkish participants (11 males, and 16 females) aged 27.6 ± 1.69 yrs, a significant decrease in body mass, body mass index (BMI), and fat-free mass. Similarly, Mengi Çelik et al. (59) reported a decrease in body mass and BMI during Ramadan in 32 (12 males 20 females) Turkish healthy adults aged 19 and 32 years. Similar findings were reported in 49 Indonesian healthy adults (15 males, 34 females) (60). Hasan et al. (61) found that Ramadan fasting decreased BMI and waist circumference in 55 overweight and obese participants (22 females and 33 males). Alsowaid et al. (62) enrolled only female individuals (*n* = 20) who were healthy students at the University of Bahrain. The authors found that Ramadan fasting decreased body mass, BMI, fat mass, and waist circumference (62). The decrease in body mass, BMI, and waist circumference could be attributed to the increased utilization of stored body fat as an energy substrate and/or the decreased energy intake as a result of fasting (63). Nevertheless, decreased total water intake during the month of Ramadan, possibly leading to a state of hypohydration, may also explain the decrease in body mass and BMI (63, 64).

Recently, Al-Maiman et al. (65) evaluated the effects of Ramadan fasting on anthropometric parameters of patients with type 1 (*n* = 42) and type 2 (*n* = 62) diabetes from Saudi Arabia, aged ≥ 20 years, and who fasted a minimum of 15 days during Ramadan. The authors concluded that Ramadan fasting decreased body mass and BMI in patients with type 2 diabetes. A significant decrease in body mass and BMI, with no significant effect of Ramadan fasting on body fat, visceral fat, and muscle mass was observed in Saudi patients (*n* = 20) with chronic kidney disease (CKD) (66). In contrast, Oueslati et al. (67) found, in 55 Tunisian type 2 diabetes (mean age=54.5 ± 10.1 yrs) who fasted 29.3 ± 2.3 days during the month of Ramadan, a significant decrease in body mass, BMI, waist circumference, fat body mass. The body mass loss was significantly correlated with the number of fasting days (*r* = 0.348, *p* = 0.009).

Badran et al. (68) found, in 98 patients with non-alcoholic fatty liver disease, that Ramadan fasting decreased significantly body mass and BMI, while waist circumference, hip circumference, waist/hip ratio, and triceps skin fold thickness remained unchanged.

It should be acknowledged that there are cases where body mass and body mass index may increase during the month of Ramadan, likely as a result of excess intake of fat, sugar, and energy, along with increased sedentariness (69).

#### Hydration status

Optimal hydration status is beneficial for physical and cognitive functioning, as well as health (70). Fluid restriction during daylight hours of the month of Ramadan may influence the hydration status of fasters. Indeed, previous reports have indicated that total water intake was less during Ramadan compared to before Ramadan (71–73), possibly leading to altered kidney function. Fortunately, fluid balance can be maintained by maximizing urinary concentration and decreasing obligatory urine output (74, 75). However, Leiper et al. (76) reported a prolonged state of hypohydration induces a degree of stress on kidney function, which may negatively affect the efficiency of the water-conserving mechanism. Hydration status can be assessed using urine (e.g., urine osmolality, urine-specific gravity) or blood (e.g., plasma/serum osmolality) markers, among others, such as the estimation of the total body water (64).

Ramadan et al. (77) evaluated the effect of Ramadan fasting on hydration status, assessed using plasma osmolarity, in 13 healthy Kuwaiti adults who were either sedentary (*n* = 7) or physically active (*n* = 6). The authors found plasma osmolarity increased significantly in sedentary individuals, suggesting a state of hypohydration (77). However, plasma osmolarity remained unchanged in the physically active group, possibly explained by an adequate water intake after the break of the fast (77).

Ibrahim et al. (73) found, in 18 healthy men (mean age = 22.0 ± 2.4 yrs), a significant increase in urine specific-gravity measures, with mean values higher than 1.020, indicative of a state of hypohydration during Ramadan. Conversely, serum osmolality levels decreased significantly during Ramadan, but remained within normal ranges. It appears the homeostatic mechanisms may have kept serum osmolality at normal levels (73). The lack of change in urine osmolality during Ramadan was also observed in 16 male Sudanese students aged 20–22 years (78). Husain et al. (79) found, in 21 Malaysian healthy individuals (12 males and 9 females), that water intake and urine output volumes during the fasting and non-fasting control periods were approximately the same. Again, the authors concluded fluid balance was maintained throughout the month of Ramadan (79).

Only three studies, with mixed findings, evaluated the effects of Ramadan fasting on the hydration status of patients. While Dikme and Dikme (80) reported the absence of the effect of Ramadan fasting on serum osmolality in 62 Turkish patients admitted to the emergency department, Azwany et al. (81) showed a significant increase in urine osmolality at the end of Ramadan vs. pre-Ramadan. In the latter study (81), urine osmolality remained within normal ranges. In patients (*n* = 31) with CKD (grades 2–4), Hassan et al. (82) found a reduction in the total body water as estimated using an impedancemeter, suggesting a state of hypohydration.

Collectively, information obtained to date does not suggest hydration status is adversely affected by Ramadan fasting. Moreover, changes in urine/plasma biomarkers are usually relatively small, with values remaining within the normal reference range. It appears fasting during Ramadan can be safely maintained for healthy individuals; however, future studies on patients with underlying chronic conditions are warranted.

#### Renal function

Optimal kidney function is of paramount importance for wellbeing and health (83). Creatinine clearance and the plasma/serum concentrations of creatinine, urea, and uric acid are all potential measures of renal function (84). Increases in serum urea and creatinine have been observed in sedentary men during Ramadan (77). However, possibly because of adaptations of renal function, the serum uric acid concentration is lower in physically active than in sedentary men (77). Other studies on healthy individuals have reported slight modifications in the levels of renal function markers, albeit with no clinical significance (85–87). To the best of our knowledge, no previous study has reported impairment of renal function in healthy individuals as a consequence of Ramadan fasting. Nevertheless, the impact of Ramadan fasting on renal function in patients with CKD is a subject of debate.

The first published systematic review evaluating the effects of Ramadan on renal function in patients with CKD was conducted by Bragazzi (38). After identifying and summarizing the results of 26 studies published between the years 1989 and 2013, the author concluded that recipients of kidney allograft can safely fast during the month of Ramadan (38). However, evidence regarding safety in patients with nephrolithiasis and CKD is equivocal. In addition, the authors pointed out the scarcity of information about Ramadan fasting falling in hot seasons (38). Finally, given the lack of evidence-based guidelines and protocols, which correctly address the issue of the impact of Ramadan fasting on CKD patients, the authors recommended the publication of randomized clinical trials.

In his systematic review and meta-analysis, Bragazzi (39) concluded patients with CKD can safely fast during Ramadan because glomerular filtration rates (GFR) do not significantly change. Additionally, sensitivity analyses revealed no impact of seasonality (39).

Other research articles have been published in an effort to better understand and evaluate the effects of Ramadan fasting in CKD patients. Mbarki et al. (88) evaluated the renal function in 60 CKD patients who fasted for the entire month of Ramadan. The authors found that 11.7% of CKD patients had an increase of serum creatinine by 442.1 μmol/L on the initial serum creatinine level, and a 25% reduction of GFR, indicating the development of acute renal failure (ARF). The study by NasrAllah and Osman (89) showed compared to non-fasting CKD patients (control group), there were significant adverse effects in the fasted CKD patients [estimated GFR (eGFR) = 27.7 mL/min/1.73 m^2^] and an increase of 60.4% in the serum creatinine after 1 week of Ramadan fasting. In addition, 3 months after the end of Ramadan, plasma creatinine remained high in 23% of the fasting CKD patients, with no significant difference vs. the non-fasting CKD patients' control group (89). The authors concluded the increase in creatinine levels was likely due to CKD progression, opposed to fasting (89). In a prospective study that enrolled 65 CKD (stage 3) patients, an increase of serum creatinine by ≥ 26.5 μmol/L in 33% of patients was reported (90). Chowdhury et al. (91) found in 68 CKD stage 3 diabetic patients who fasted ~19 h, a significant difference in proteinuria and ARF risk compared to 61 same-category patients who did not fast.

A significant improvement in eGFR (from 29.7 mL/min/1.73 m^2^ to 32.7 mL/min/1.73 m^2^ after fasting) in diabetic individuals was reported (92). Despite non-optimal hydration status and decreased serum basal B-type natriuretic peptide, Hassan et al. (82) found no significant differences in eGFR between fasting and non-fasting CKD patients (stages 2-4). In Turkish patients (45 fasters and 49 non-fasters CKD, stages 3-5), Kara et al. (93) found no significant changes in eGFR between non-fasters and fasters; however, individuals above the age of 72 appeared to be at a higher risk of renal function impairment than CKD patients under the age of 64. Recently, a retrospective study of 1,199 patients, who were not exempt from Ramadan fasting for 2 years (2016 and 2017), showed fasting significantly reduced the risk of developing ARF, particularly in patients with comorbidities (94). The authors concluded that Ramadan fasting conferred no negative effects on the majority of patients with comorbid disorders (94). Similarly, following Ramadan fasting, no significant impairment in renal function was found in autosomal dominant polycystic disease patients with early CKD (95). Baloglu et al. (96) found that hypertension and fasting days during Ramadan are strong predictors of ARF, where ARF developed in 27/117 patients with stage 2-3 CKD, with an average age of 60 years. The authors recommended adequate hydration and regular check-ups in order to lower the risk of developing ARF during Ramadan (96). According to an Egyptian-based study (97), serum creatinine increased significantly after Ramadan fasting, although eGFR remained unchanged in individuals without CKD. Conversely, serum creatinine in CKD patients was lowered, and eGFR improved significantly during Ramadan, most likely as a result of better blood pressure control in hypertensive CKD patients (97).

In summary, Ramadan fasting appears to have no adverse effect on the renal function of healthy individuals. However, findings in CKD patients are mixed and it is difficult to determine the reasons for discrepancies in the identified studies. The differences between the studied populations in terms of CKD severity, hydration levels during non-fasting hours, fasting days, fasting length, observation period, and other lifestyle factors such as physical activity level may explain the contradictory findings. Fasting during the month of Ramadan tends to be more harmful in patients with increasing degrees of renal impairment; however, this is not universally true in all aforementioned papers. Recently, Malik et al. (98) recommended that stable CKD non-dialysis (up to Stage 3) patients may be able to fast with close monitoring, while hemodialysis and peritoneal dialysis patients are considered very high risk.

#### Metabolic health

Metabolic health is a broad term covering numerous elements of cellular, cardiovascular, and cardiorespiratory health and wellbeing (99). Anthropometrics, blood pressure, and blood-based indices, such as blood glucose and serum lipids can all be utilized in clinical settings to assess metabolic health (99). One well-established method is to use diagnostic criteria of metabolic syndrome (MetS) (100), a multidimensional disorder that predisposes people to serious health concerns such as atherosclerotic heart disease (101) and type 2 diabetes (102).

Kul et al. (23) conducted the first systematic review and meta-analysis evaluating the effects of Ramadan fasting on blood levels of lipids and fasting glucose, considering gender differences. The authors found that compared to pre-Ramadan levels, fasting during Ramadan significantly decreases LDL-cholesterol and fasting blood glucose levels in both sexes (23). In females, total cholesterol (TC) and triglyceride (TG) levels did not change, while HDL-cholesterol levels increased during vs. before Ramadan. In males, there was a significant decrease in total cholesterol, LDL-cholesterol and TGs.

The previous systematic review and meta-analysis was updated by Mirmiran et al. (26). The authors concluded that Ramadan fasting has no significant effect on circulating triglycerides, total cholesterol and LDL-cholesterol in apparently healthy individuals. Conversely, Ramadan fasting results in decreased levels of HDL-cholesterol and very low-density lipoprotein cholesterol (VLDL-C), as well as increased LDL-cholesterol levels.

Faris et al. (28) evaluated the effect of Ramadan fasting on glucometabolic markers (i.e., fasting glucose, insulin, insulin resistance, leptin, adiponectin) in healthy non-athletic individuals. The authors concluded that fasting during Ramadan decreases significantly fasting glucose, with a minimal non-significant effect on insulin, insulin resistance, leptin, and adiponectin. The authors concluded Ramadan fasting has no adverse metabolic impacts, and could help improve glucometabolic markers, particularly fasting glucose levels, in healthy individuals.

Faris et al. (27) conducted a meta-analysis evaluating the effect of Ramadan fasting on MetS components among healthy Muslims. Results revealed that Ramadan fasting decreases significantly waist circumference, fasting blood glucose, serum triacylglycerol and systolic blood pressure, while it increases HDL-cholesterol (27).

Recently, Jahrami et al. (29) conducted a meta-analysis to evaluate the effect of Ramadan fasting on cardiometabolic risk factors in healthy adults. The authors found that Ramadan fasting significantly decreases total cholesterol, LDL-cholesterol, VLDL-C, TGs and diastolic blood pressure, while it increases HDL-cholesterol (29). Resting heart rate did not change during compared to before Ramadan (29). The authors concluded Ramadan fasting positively impacts cardiometabolic risk factors, which may provide healthy persons short-term protection against cardiovascular diseases (29). The beneficial effect of Ramadan fasting on systolic and diastolic blood pressure was originally proposed in the meta-analysis by Al-Jafar et al. (36).

The effect of Ramadan fasting on the main hormones regulating appetite and satiety (i.e., leptin, and adiponectin) was investigated in a systematic review and meta-analysis by Gaeini et al. (44). Accordingly, a significant decrease in leptin levels was observed after Ramadan fasting. Ramadan fasting had no significant effect on the levels of adiponectin. In addition, the sub-group analysis demonstrated a greater decrease in leptin levels among normal-weight individuals compared to those of overweight/obese subjects. The authors concluded Ramadan fasting may decrease leptin levels, particularly in normal-weight individuals.

The beneficial effects of Ramadan fasting on glycaemic parameters in patients with type 2 diabetes was also shown by Aydin et al. (24). In their systematic review and meta-analysis, the authors found Ramadan fasting significantly decreases fasting plasma glucose (24). Additionally, the subgroup analysis revealed the decrease in fasting blood glucose was observed in the monotherapy (single oral antidiabetics) group, the oral combination therapy (multi oral antidiabetics) group, and the multi-treatment (oral antidiabetics plus insulin or diet modification) group (24). However, postprandial plasma glucose, glycated hemoglobin, and fructosamine levels remained unchanged during vs. before Ramadan (24). BMI decreased significantly in the oral combination therapy group alone (24). The authors concluded Ramadan fasting has no significant negative effects on postprandial plasma glucose and fructosamine levels. Interestingly, BMI and fasting plasma glucose were positively impacted by Ramadan fasting (24). Other recent empirical studies reported improvement in the metabolic profile in diabetics during Ramadan (103, 104).

In summary, Ramadan fasting improves the metabolic health of healthy individuals and type 2 diabetes patients. However, according to an epidemiological study of 13 countries with large Muslim populations in northern Africa, Asia, and the Middle East, hypoglycaemic episodes increased in people with diabetes (types 1 and 2) during Ramadan (105). Fortunately, the results of the systematic review and meta-analysis of Tahapary et al. (25) showed patients with diabetes who fasted during Ramadan had a low incidence of hypoglycemia and no studies reported fatal hypoglycemic events. Supporting this, Almulhem et al. (35) concluded there is insufficient evidence to link Ramadan fasting to an increased or decreased risk of cardiovascular events in diabetics. It is worth noting that Ramadan is challenging when it falls close to the summer (when daylight hours are longer) (106). Thus, contemporary recommendations for patients with diabetes seeking to participate in fasting during Ramadan should be followed (106, 107). Recent guidelines, authored by the International Diabetes Federation and the Diabetes and Ramadan International Alliance (IDF-DAR), pertaining to the management of patients with diabetes during the month of Ramadan have been developed (107). In brief, a health assessment, pre-Ramadan, should be conducted, taking place around 6–8 weeks prior to commencing Ramadan. This approach would permit health care professionals to obtain a detailed medical history, in addition to performing a risk assessment, thus facilitating the classification of diabetic patients into a low-risk (i.e., fasting is probably safe), moderate-risk (i.e., fasting safety is uncertain), or high-risk (i.e., fasting is probably unsafe) group (107). This classification system will underpin all subsequent recommendations, which will include guidance related to the safety of fasting, strategies/approaches for modifications or adjustments in dosage or treatment regimen, Ramadan-focused education, and dietary guidance (107). Following the above steps, individuals who elect to fast must adhere to diabetes management guidelines during Ramadan fasting, inclusive of changes to glycemic monitoring schedules and adjustments in medication dosing. Finally, a follow-up post-Ramadan is advised. This will assist healthcare professionals in identifying and understanding critical information about the individual's Ramadan successes and challenges, ensuring that subsequent participation in Ramadan fasting is more successful (107). Moreover, this process must be replicated each Ramadan because fasting safely at one-time point does not guarantee the same level of success the following year (107).

It should be acknowledged that utilizing analogs of basal insulin during Ramadan is recommended due to the relatively lower risk of hypoglycemia, as compared to regular human insulin (108, 109). For instance, for once-daily basal insulin analogs, the IDF-DAR guidelines advocate a reduced (15–30%) dose, administered at the break of fast (i.e., *iftar*) (107). Further, recent empirical evidence has highlighted the safety and effectiveness of insulin glargine 300 U/mL, a second-generation basal insulin analog, in patients with type 2 diabetes who fasted during Ramadan (110, 111). Further specific recommendations for type 1 and type 2 diabetics who observe Ramadan are also provided in the recent guidelines (107).

Structured diabetes education is important for diabetic patients who will observe Ramadan (112, 113). Previous studies concluded diabetic patients who benefited from a structured diabetes education (i) improved their blood glucose levels/glycemic control (112, 113), (ii) reduced the incidence of hypoglycemic events (112, 113) and hyperglycemic crises (113), (iii) decreased their body mass (112), and (iv) increased their acceptance and frequency of glycemia measurements (113). Interestingly, Bravis et al. (112) reported glycated hemoglobin reduction was sustained 12 months after attending the structured diabetes education.

The Diabetes and Ramadan International Alliance recommended practicing regular light-to-moderate exercise during Ramadan for patients with diabetes (114). In that regard, *Attarawih* prayer, which includes movements such as kneeling, bowing and rising, should be included in the daily exercise routine of diabetic patients (114).

Public health authorities should prioritize structured diabetes education for patients with diabetes who will fast during the month of Ramadan.

#### Liver function

The liver is a vital organ with multiple functions ranging from detoxification of drugs and toxic chemicals, regulation of red blood cells, and maintaining energy metabolism and bile acid homeostasis (115). The liver function tests typically include alanine transaminase (ALT) and aspartate transaminase (AST), alkaline phosphatase (ALP), gamma-glutamyl transferase (GGT), serum bilirubin, prothrombin time (PT), the international normalized ratio (INR) and albumin (116).

Faris et al. (37) conducted a systematic review and meta-analysis to evaluate the effects of Ramadan fasting on liver function tests in healthy people, and to examine the impact of different covariates using subgroup analysis and meta-regression. Faris et al. (37) found that Ramadan fasting induced a significant positive effect on markers of liver damage including AST, ALP, and bilirubin. The authors concluded Ramadan fasting could confer short-term protection against liver steatosis in healthy individuals (37), possibly attributed to the improvement in the metabolic profile (e.g., increased HDL-cholesterol and decreased TC and LDL-cholesterol) and the decrease of body mass, waist circumference and body fat percentage (37). However, the small number of included studies, particularly those assessing GGT, warrant further research.

The effects of Ramadan fasting on salivary liver function tests were investigated by Besbes et al. (30) who concluded salivary ALP increased, whilst AMP decreased significantly during vs. before Ramadan.

Recently, the positive effect of Ramadan fasting on liver function in 70 healthy Iranian individuals was reported (117). Similar to healthy individuals, Ramadan fasting improved non-invasive measures for non-alcoholic steatohepatitis severity assessment (118, 119). Furthermore, it appears to have no negative effect on the liver function of obese males (120).

Collectively, Ramadan fasting appears to protect liver function in healthy individuals and improves non-invasive measures in patients with non-alcoholic steatohepatitis. However, more rigorous studies on patients are needed during Ramadan.

#### Markers of inflammation, immunity and oxidative stress

The imbalance between the generation of reactive oxygen species and the availability of antioxidants or radical scavengers results in oxidative stress (121). Excess reactive oxygen species can either oxidize biomolecules or structurally change proteins and genes, resulting in signaling cascades that can contribute to inflammatory-related diseases (121).

It well-known that patients with chronic diseases are exempt from fasting during Ramadan. However, while some of these patients are eager to celebrate this time of year with their peers, there are no guidelines to assist physicians in dealing with the concerns of those with infectious diseases who choose to fast during Ramadan. In their systematic review, Bragazzi et al. (31) concluded that (1) patients with diabetes who are at risk of developing infectious complications should not fast, (2) Ramadan fasting has little effect on diarrheal patients (31), (3) HIV is a challenge, and patients should be advised to take *ad hoc* drug combinations, and to not eat fatty meals that could interfere with the treatment (31), (4) Ramadan has no effect on anti-helminthic therapy effectiveness (31), and (5) patients with active ulcers should not fast, because they are more likely to develop complications (31).

The second systematic review on the topic was conducted by Adawi et al. (32). The authors synthesized 45 studies and found that: (i) Ramadan fasting can mildly influence the immune system, with generally transient alterations, returning to baseline pre-Ramadan values immediately afterward; (ii) Ramadan fasting during the second trimester of pregnancy is safe, not resulting in negative fetal outcomes or maternal oxidative status alterations; (iii) Ramadan fasting can enhance lipid profile and mitigate against oxidative stress in cardiovascular patients; (iv) Ramadan fasting is safe among asthmatic patients as well as in patients with HIV/AIDS and autoimmune disorders, and (v) Ramadan fasting can lead to increased immunological markers in psychiatric patients.

Faris et al. (33) performed a systematic review and meta-analysis to examine changes in inflammatory and oxidative stress markers in healthy individuals before and after Ramadan. The authors found that fasting during Ramadan resulted in very small reductions in interleukin (IL)-1, C-reactive protein (CRP)/high sensitivity-CRP, and malondialdehyde (MDA), and small reductions in tumor necrosis factor-α and IL-6. Faris et al. (33) concluded that fasting during Ramadan protects healthy people against elevated inflammatory and oxidative stress markers. This could be attributed to the reduction caloric intake, and the associated decrease in body mass (predominately body fat).

In their systematic review, Besbes et al. (30) concluded that salivary immunoglobulin-A, playing an important role in mucosal immunity, decreased during the last week of Ramadan. It is worth noting that because there is no salivary IgA concentration threshold (122), a decrease in those levels does not necessarily imply that the participant is more susceptible to oral infection onset.

In obese and overweight subjects (*n* = 114), Makdour et al. (123) showed that Ramadan fasting ameliorates the genetic expression of antioxidant and anti-inflammatory and metabolic regulatory genes, which may confer protection against oxidative stress and its metabolic-related disorders in non-diabetic obese individuals. Compared to non-fasting obese males (*n* = 14), improvement in systemic inflammation biomarkers in obese males fasting during Ramadan (*n* = 14) has been reported (120).

#### The gut microbiome

The human gastrointestinal microbiome, which contains millions of organisms (e.g., bacteria, viruses, fungi, parasites), can be influenced by various environmental factors (e.g., diet). Conversely, various studies have shown that adverse changes in the intestinal microbiome can be associated with the development of various chronic diseases such as metabolic diseases (124), liver diseases (125, 126), neurodegenerative disorders (127), cancer (128), and others. Some findings have revealed that fasting diets can also cause changes in the microbiome (129, 130). In a recent systematic review, Mousavi et al. (43) reported that Ramadan fasting improves health parameters through positive effects on some bacterial strains such as *Akkermansia muciniphila* and *Bacteroide*.

The positive effects of Ramadan fasting on gut microbiota were recently demonstrated by Chen et al. (131). The authors investigated the influence of fasting during Ramadan on the gut metabolic profiles in fecal samples obtained from healthy Chinese (*n* = 16) and Pakistani (*n* = 18) subjects, using liquid chromatography-mass spectrometry-based metabolomics analysis (131). Chen et al. (131) concluded fasting leads to changes in metabolite profiles specific to each ethnic group in a manner dependent on dietary components. Additionally, these changes are correlated with dynamic shifts in microbiota composition and diversity which, in conjunction with dietary changes during Ramadan fasting, lead to the enrichment or depletion of various functional metabolites (131).

Another study showed that Ramadan fasting resulted in significant beta diversity and enrichment in the *Bacteroidetes phylum* (132). The increase in the *Bacteroidetes phylum* after Ramadan fasting is important since a decrease in the *Bacteroidetes*/*Firmicutes* ratio has been found to play a key role in the development of obesity (133).

Su et al. (130) concluded that Ramadan fasting provokes substantial remodeling of the gut microbiome in healthy non-obese individuals and that this remodeling involves the upregulation of *Lachnospiraceae* species. It should be acknowledged that *Lachnospiraceae* and *Ruminococcaceae* are both bacteria that produce butyric acid, which helps to reduce oxidative stress, inflammation, and the risk of colon cancer (134–136).

In summary, Ramadan fasting appears to be beneficial for the gut microbiome of healthy individuals; however, future studies on patients are warranted.

#### Mental health

Good mental health is defined as a state of wellbeing that allows a person to cope with the normal stresses of life and function productively (137). Prevention of mental disorders (e.g., bipolar disorders) has emerged as an essential component of modern clinical psychiatry (137). Mental health can be assessed using psychometric interviews or specific questionnaires (137). One systematic review and meta-analysis evaluated the effects of Ramadan fasting on mental health in individuals without psychiatric disorders (45). The authors concluded that Ramadan observance was associated with lower stress, anxiety, and depression (45). However, given the small number of included studies (*n* = 5), the findings of Berthelot et al. (45) should be interpreted with caution. Additionally, the authors reported that no previous studies were carried out in psychiatric patients and recommended further studies should include these populations (45).

Current evidence suggests Ramadan fasting may not be considered a non-pharmacological therapy enhancing the mental health of healthy individuals, but promising results exist.

#### Sleep

Sleep plays a vital role in maintaining good overall health (138). Prolonged sleep loss is a risk factor for the development of non-communicable diseases (NCDs) such as diabetes, obesity, hypertension, heart disease and stroke and may contribute, in the long term, to premature death (139).

To date, the systematic review and meta-analysis of Faris et al. (46) is the only to estimate the effect size of Ramadan fasting on sleep duration and daytime sleepiness. The results revealed sleep duration decreased from 7.2 h per night before Ramadan to 6.4 h during Ramadan, while the Epworth sleepiness scale score, indicative of daytime sleepiness, increased slightly from 6.1 before Ramadan to 7.0 during Ramadan. Therefore, Faris et al. (46) concluded Ramadan fasting decreases sleep duration and increases daytime sleepiness levels. The increased night-time social activities (e.g., meetings in the coffee, prayer such as *Attarawih*, Quran reading group) and the large amount of food consumed after the break of fast, possibly delaying sleep, may explain these findings.

It should be acknowledged that almost all studies assessed sleep parameters using subjective tools. Therefore, the previous results should be interpreted with caution.

Recently, Mengi Çelik et al. (59) assessed sleep quality before and during the month of Ramadan, using the Pittsburgh Sleep Quality Index Questionnaire, in 32 (12 males 20 females) healthy Turkish adults. The authors found no significant effect of Ramadan fasting on sleep quality in healthy individuals. A similar result was observed in female healthy students from Bahrain (62). Alzhrani et al. (140) reported that sleep duration remained unchanged from before to during Ramadan in 115 adults (96 females and 19 males) form Saudi Arabia.

Collectively, Ramadan fasting appears to negatively affect sleep parameters. Future studies, with higher methodological rigor, evaluating strategies (e.g., sleep hygiene education, supplementation) aiming at improving sleep assessed using objective tools (e.g., polysomnography) are warranted.

#### Pregnancy outcomes

Despite being exempt, many pregnant Muslim women choose to fast during Ramadan. However, a sub-optimal diet during pregnancy could impact birth weight. Glazier et al. (47) investigated whether Ramadan fasting by pregnant women affects perinatal outcomes including perinatal mortality, preterm birth and small for gestational age infants, stillbirth, gestational diabetes, hypertensive disorders of pregnancy, congenital abnormalities, serious neonatal morbidity, birth weight, preterm birth, placental weight, neonatal death and maternal death. The authors concluded Ramadan fasting had no negative effects on birth weight, but there is insufficient evidence on potential effects on other perinatal outcomes (47). As a result, future studies on this topic are needed.

## Greek orthodox Christian fasting

The Christian Orthodox Church (COC) recommends that those who follow a long-term organized fasting diet must refrain from meat, dairy products, and eggs for 180–200 days every year while increasing their consumption of grains, fruits, legumes, vegetables, fish, seafood and olive oil. COC fasting consists of three main fasting periods: 40 days before Christmas (from 15 November to 24 December), 48 days before Easter (from Clean Monday to Holy Saturday), 14 days before Assumption (from 1 to 14 August), the fasting period before Holy Apostles (lasting 0-30 days depending on Easter feast), and three other daily feasts (5 January, 29 August, 14 September), in addition to every Wednesday and Friday (141).

### Food choices

The COC fasting regime could be described as a periodical vegetarian diet, in which fish are occasionally and seafood always permitted (142), sharing numerous similarities with the typical Mediterranean diet (21, 143, 144). Therefore, it is not surprising previous studies reported increases in the consumption of fruits, vegetables and legumes in fasting individuals according to COC recommendations (142, 143, 145–147).

### COC fasting and health-related indices

One systematic review (21) and one scoping review (22) evaluating the effects of COC fasting on health-related indices has been published. Interestingly, almost all included studies in those reviews were conducted in Greece.

Koufakis et al. (21) concluded that COC fasting has a beneficial effect on the lipid profile, with the decrease of TC and LDL-cholesterol levels being a consistent finding across trials (i.e., up to 17.8 and 31.4%, respectively). This was previously attributed to the decrease in the intake of energy and saturated fatty acids (148–150). However, the impact of COC fasting on HDL-cholesterol is controversial (21), with some studies reporting a significant decrease in HDL-cholesterol (151, 152), whilst others reported a lack of change (143, 153). It should be acknowledged that the decrease in HDL-cholesterol was previously observed after vegetarian diet intervention (154).

Conclusions on the influence of COC fasting on body mass, glucose homeostasis, blood pressure, antioxidant factors, iron status and relative hematological parameters cannot be drawn since relevant evidence is scarce and/or yields contradictory findings (21). Koufakis et al. (21) noted that any potential negative effect of COC fasting, primarily due to the reduced intake of vitamin D and B12 and minerals (mostly calcium), warrants additional investigation. Additionally, the authors pointed out that incorporating this dietary pattern on a daily clinical basis as a health-promoting diet should be rigorously appraised and individually assessed, because current evidence is inconclusive, necessitating further investigations (21).

In their recent scoping review, Kokkinopoulou and Kafatos (22) suggested a beneficial effect of COC fasting on the lipid profile, marked by a decrease in total cholesterol and LDL-cholesterol. Additionally, the authors showed COC fasting reduced body mass, BMI and systolic blood pressure. Therefore, The COC fasting diet pattern may be recommended for the prevention of chronic diseases, as well as for persons who desire to adopt a plant-based diet for a healthier and/or more sustainable way of life (22). Although there is evidence in favor of health advantages, the COC fasting dietary recommendations, similar to any other dietary patterns, should always be followed under individualized supervision on effective meal planning (22). Nevertheless, long-term follow-up studies should be conducted to assess whether the favorable impacts of COC fasting on health markers are maintained over time (22).

A recent study by Kokkinopoulou et al. (155) investigated the relationship between COC fasting recommendations and cancer risk, specifically focusing on fiber, vegetables, fruit, and red and processed meat consumption. The authors found the diet of fasters to be healthy and follows the World Cancer Research Fund Cancer Recommendations (156), which is in favor of decreasing the chance of developing colorectal cancer compared to their non-fasting counterparts. Furthermore, eating more vegetables and fruits and eating less overall processed meat could reduce the risk of MetS.

In another study (157), plasma adiponectin, biochemical, and anthropometric data were collected from 55 COC fasters and 42 time-restricted eating (TRE) controls (all women, mean age = 47.8 years) at three different time points: baseline, the end of the dietary intervention (7 weeks), and 5 weeks after participants resumed their usual dietary habits (12 weeks from baseline). In the COC fasting group, adiponectin levels increased significantly at 12 weeks compared to baseline, and body fat mass decreased significantly between baseline and 12 weeks and between 7 and 12 weeks. Throughout the investigation, an inverse relationship between adiponectin and waist circumference values was detected in the same group. The authors concluded that COC fasting has beneficial metabolic effects linked to improved adiponectin levels.

Other recent studies showed the beneficial effect of COC fasting on metabolic health indices (148, 149, 158). For example, it has been shown that COC fasting has superior lipid-lowering effects compared to the TRE pattern in overweight adults (148). In addition, the favorable long-term effects of COC on irisin levels (involved in the adipose tissue browning) in overweight, metabolically healthy, adults have been shown (158).

Interestingly, in the study of Papazoglou et al. (16), participants were 60 Greek Orthodox volunteers (30 with dyslipidemia and 30 without) who followed the COC fasting for 7 weeks, and 15 young (non-dyslipidemic) Muslim individuals who observed Ramadan fasting. The serum blood tests of study participants were measured pre- and post-fasting for biochemical (iron, ferritin, vitamin B12, calcium, LDL-cholesterol, HDL-cholesterol, TC, TG, and fasting glucose) and hematological (hemoglobin, hematocrit) (16). The results showed that COC fasting resulted in significant decreases in fasting glucose, HDL-cholesterol, LDL-cholesterol, and TC in both dyslipidemic and non-dyslipidemic Orthodox individuals. Hemoglobin, hematocrit, iron, and ferritin levels were significantly higher after COC fasting, although vitamin B12 and calcium levels were significantly lower (16). Further, a subanalysis of dyslipidemic and non-dyslipidemic Orthodox individuals found the former had a greater decrease in cholesterol levels (16). While TG, LDL-cholesterol, and TC levels were all higher in Muslim participants after Ramadan fasting, no significant effect was observed for the remaining assessed blood-based health indices (16). The authors concluded that prevention of calcium and vitamin B12 deficiency during COC fasting by supplement consumption is needed.

The sex-specific changes in lipid concentrations during COC fasting, as well as the potential role of vitamin D status in mediating these variations, have been investigated in a 12-week prospective intervention (159). In this study, biochemical data on serum lipids and vitamin D status were collected at baseline, 7 weeks after COC fasting, and 5 weeks after fasters resumed their standard dietary habits (12 weeks from baseline) (159). Based on 25-hydroxyvitamin D [25(OH)D] concentrations, participants (24 premenopausal females, 53%) were divided into two groups: those with concentrations above and below the median values (159). The study's findings revealed sex-specific differences in the pattern of lipid profile changes following COC implementation (159). Additionally, female participants with 25(OH)D concentrations below the median at each time point showed variability in TC and LDL-cholesterol responses; a 15% reduction was observed during the fasting period, followed by an appreciable increase slightly above baseline values 5 weeks after OF cessation (159). A similar pattern, albeit less pronounced, was observed for HDL-cholesterol concentrations, as well (159). For male participants, an inverse non-significant trend of TC, LDL-cholesterol, and HDL-cholesterol increase during the study period was observed; however, this was only seen in individuals with 25 (OH)D concentrations below the median (159).

Athonian COC fasting, practiced by Athonian monks, is a pescetarian variation of the typical COC fasting (160). Athonian monks must abstain from red meat consumption throughout the year, during both fasting and non-fasting periods (160). Karras et al. (160) showed lower BMI, body fat mass, and homeostatic model assessment-insulin resistance in male Athonian monks (*n* = 57), compared to a general population practicing the typical COC fasting (*n* = 43). Additionally, the authors found that secondary hyperparathyroidism, resulting from profound hypovitaminosis D, was observed in the Athonian monks' group.

Nonetheless, larger sample size studies and/or long-term follow-ups are warranted before drawing firm conclusions on the effects of COC fasting on human health.

The effect of COC fasting on cognitive function and emotional wellbeing of healthy adults was evaluated by Spanki et al. (161). Two groups of fasting (*n* = 105) and non-fasting (*n* = 107) individuals were evaluated regarding their cognitive performance using the Mini Mental Examination Scale and the presence of anxiety and depression using, the Hamilton Anxiety Scale, and the Geriatric Depression Scale, respectively. The authors concluded that COC fasting has a positive effect on cognition and mood in middle-aged and elderly individuals (161).

There is a growing body of evidence highlighting the beneficial effects of COC fasting on the health of Orthodox individuals. Unfortunately, studies evaluating the effects of COC fasting in patients are scarce. Future COC fasting-based studies in diverse ethnic populations, pregnant and breast-feeding women, and patients are also warranted.

## Other types of religious fasting

### Health-related indices

#### The Daniel fast

The Biblical-based Daniel fast, practiced by some Christians, prohibits the consumption of animal products, refined carbohydrates, preservatives, food additives, flavorings, sweeteners, alcohol, and caffeine (162). It is typically observed for 21 days, while fasts of 10 and 40 days have been reported (162).

Bloomer et al. (163) evaluated the effect of Daniel fast on human metabolic and cardiovascular health in 43 healthy individuals. The authors found that total energy, protein, fat, saturated lipids, trans-fat and dietary cholesterol intake decreased while carbohydrates, fibers, and vitamin C intake increased. They also found significant reductions in TC, LDL-cholesterol, HDL-cholesterol, systolic blood pressure, and diastolic blood pressure.

The effect of Daniel fast on glucose homeostasis has also been investigated (164). Following the Daniel fast, a significant decrease in glycemia, fasting blood insulin and HOMA-IR was observed (164). Interestingly, the Daniel fast appears to decrease blood oxidative stress (as measured by MDA), increase antioxidant capacity (as measured by TEAC), and increase nitrate/nitrite levels (165). The improved antioxidant status was attributed to a combination of lower calorie and saturated fat intake and an increase in nutrient- and fiber-rich fruit, whole grain and vegetable consumption (165). In addition, the elimination of dietary additives, preservatives, and processing agents, as well as a reduction in protein consumption (methionine included) may explain the beneficial effect of the Daniel fast on the oxidative stress profile (165).

Unfortunately, given the scarcity of Daniel fast-related studies, no firm conclusions can be drawn on its health impacts.

#### The Jewish Yom Kippur fast

Yom Kippur occurs on the 10th day of the 7th month of the Hebrew calendar, after Rosh Hashanah (the Jewish New Year), and is distinguished by a prohibition on eating and drinking (166). The Jewish Yom Kippur fast differs from other fasting rituals in that it requires full abstinence from food and water for 25 h (167). The fast begins before sunset the evening before Yom Kippur and finishes after midnight the next day (166). It should be acknowledged that on Yom Kippur, ingestion of a shiur (~ a half mouthful of liquid) is allowed at intervals of between 4- and 9-min, and Jewish fasters can consume (if necessary) periodically 30 cc of food (MBE, 2009). This fast is short to generate major metabolic alterations leading to significant health implications (167). Unfortunately, available human studies regarding the effect of this fasting ritual on fasters' health are lacking, precluding a firm conclusion on its effects on health.

#### The Buddhist fasting

Buddhist fasting pattern, observed all year, is similar to a standard vegetarian diet that excludes meat and dairy products (occasionally milk). In addition, it is forbidden to consume five pungent vegetables (garlic, garlic chives, Welsh onion, leeks, and asant), processed foods, and alcohol (144). Food consumption varies by country and culture (168, 169). Few studies have evaluated the effects of Buddhist fasting on health-related indices. In a cross-sectional study, Ho-Pham et al. (170) evaluated the effects of a lifelong vegetarian diet on bone mineral density and body composition in a group of postmenopausal women. Participants were 105 Mahayana Buddhist nuns (vegans) and 105 omnivorous women (average age = 62, range = 50–85 yrs) (170). The results showed that although vegans consumed less calcium and protein than omnivores, veganism showed no negative effects on bone mineral density or body composition (170). Additionally, no significant adverse effects have been reported (170).

Lee and Krawinkel (168) compared body composition and nutrient intake data of 54 Buddhist vegetarian nuns and 31 omnivore Catholic nuns from South Korea. The authors found (i) nutrient intake of Korean Buddhist vegetarians was comparable to that of omnivores, and (ii) the intake of some nutrients in Buddhist vegetarians was more favorable than in omnivores (168). Additionally, Buddhist vegetarians had higher fat-free mass, body fat, and BMI than omnivores, and body fat was inversely correlated with the duration of vegetarianism for vegetarians (168). Future studies evaluating the effect of Buddhist fasting in high-risk populations are warranted.

## Religious fasting as a public health nutrition strategy

NCDs, often known as chronic diseases, are a growing concern for national governments and society throughout the world due to their high mortality and morbidity rates (171). NCDs are caused by a variety of factors, including behavioral, environmental, genetic, and physiological factors (171). They share four major behavioral risk factors: unhealthy diet, tobacco use, excess alcohol consumption, and sedentariness (172). According to current WHO projections, the total number of NCD deaths will rise to 55 million by 2030 (WHO). In 2012, all countries committed to lowering premature mortality from NCDs by 25% by 2025 (the 25 x 25 target) (173). Furthermore, the reduction in NCD premature mortality is included in the Sustainable Development Goals (SDGs), as SDG target 3.4 is to reduce NCD premature mortality one-third by 2030, compared to 2015 levels, and promote mental health and wellbeing (174). Public health practitioners and policymakers are responsible to ensure the health of all individuals by searching for and implementing effective public health strategies enabling the prevention and management of NCDs.

When examining the religious recommendations related to some types of fasting, some observations can be noticed. First, the consumption of alcohol is prohibited during Islamic, Daniel, and Buddhist fasting, and occasionally permitted during COC fasting. It is worth noting that alcohol consumption is a cause of over 200 diseases and injuries, accounting for 5.3% of premature deaths and 5.1% of the global disease and injury burden (174). Individuals strictly adhering to religious fasting will refrain from alcohol consumption, which will contribute to the improvement of their health status.

Second, during Ramadan fasting, smoking is not permitted during daylight. It should be acknowledged that tobacco smoking is the single leading cause of premature death worldwide (175), leading to the death of more than 8 million people each year (174). More than 7 million of these deaths are directly related to tobacco use (174). While adhering to Ramadan fasting will likely reduce smoking during this month (176, 177), the challenge is to maintain this habit following the break of the fast and after Ramadan (178). In this context, behavioral approaches may be effective to achieve this goal. For example, Ismail et al. (179) demonstrated a religious-based smoking cessation behavioral intervention delivered to Muslims from Malaysia during Ramadan resulted in sustained smoking reduction after Ramadan. Unfortunately, to our knowledge, no data exists on the effects of other religious fasting regimens on smoking habits.

Third, the COC fasting regimen could be described as periodic vegetarian advocacy, with several similarities to the typical Mediterranean diet and incorporating almost all of its cardioprotective and health-promoting mechanisms, such as calorie restriction and a lower intake of dietary cholesterol and fatty acids (180). Consequently, observers of COC fasting will consume a healthy diet, in which macronutrients are consumed in appropriate proportions to support energetic and physiologic needs, without excess intake, while also providing adequate micronutrients and hydration to meet the body's physiologic needs (181). Similarly, the Buddhist diet appears healthy, as it is similar to a standard vegetarian diet (144).

Unfortunately, the diet of Muslim observers (consumed after the break of fast) during Ramadan could be characterized as “unhealthy.” In this context, Khaled and Belbraouet (182) reported that Ramadan observers consume more food rich in saturated fat, sugar, and processed carbohydrates, which may mask/reduce the beneficial effects of Ramadan fasting on health. Shifting toward a healthy diet (i.e., multiple plant-based diets) such as the Mediterranean diet or the Dietary Approaches to Stop Hypertension (DASH diet) will be beneficial for the health of Muslim observers. Current evidence suggests plant-based diets reduce the risk of NCDs such as obesity, cardiovascular diseases, type 2 diabetes, and some cancers (5). Interestingly, these dietary patterns might include animal products (e.g., dairy, moderate quantities of red meat), and have all been shown to be healthier and associated with less environmental impact than the average diet in the United States (12).

Given the lack of restriction on food choices during Ramadan fasting, and the possible lack of food literacy in many Muslim individuals (183), nutritional education for Muslims before the month of Ramadan is warranted. Additionally, stakeholders involved in public health should actively participate in consumer health programs focused on nutrition awareness.

Fourth, previous studies have revealed that level of physical activity is generally reduced during fasting rituals. For example, Farook et al. (184) observed decreases in the objectively assessed habitual physical activity of adults during the month of Ramadan. Similarly, Lessan et al. (185) reported that the total number of steps walked per day were significantly lower during Ramadan. Also, Buddhists are characterized by a sedentary lifestyle; they were only participating in some “religious” daily light activities (e.g., chanting, maintenance of the temple premises, meditation) (186). However, it is well-known that persistent low levels of physical activity are detrimental to individual health and wellbeing (187, 188) and that adhering to regular a physical activity program is effective in reducing the burden of NCDs (189), as well as premature morbidity and mortality (190). Unfortunately, the results of a recent study (191) revealed religiosity did not play a mediating role in physical activity participation. Therefore, the promotion of physical activity during and outside fasting periods is needed. It should be noted that undertaking physical activity in a fasting state improves body composition (i.e., body fat) and some metabolic indices (e.g., HDL-cholesterol) (192, 193).

Finally, the frequency of emergency department admissions appears not to change (194–198) or even decrease (199) during Ramadan, suggesting no adverse effects regarding the safety of fasting during the month of Ramadan. Nevertheless, data related to the effect of other types of religious fasting on the frequency of emergency department admissions are lacking. Given the healthy diet and the possible intake of water characterizing other religious fasting regimens, it is hypothesized a decrease in the frequency of emergency department admissions will be observed.

Taken together, religious fasting regimens appear safe and beneficial for public health. Individuals involved in the public health sector such as physicians and nutritionists should encourage religious believers to adhere to fasting. Public health stakeholders should encourage public mass media to promote elements found in religious-based fasts as a nutritional public health strategy. Additionally, to optimize health gains from fasting, sessions of nutritional education (e.g., workshops, and seminars) should be organized during and outside the fasting rituals.

## Religious fasting and planetary health

The prestigious medical journal, *The Lancet*, published at the beginning of 2014 a manifesto for planetary health, which was signed by 7,390 scientists from all over the world, primarily from the fields of medicine, public health, health care, environmental science, and ecology (200). *The Lancet* declared planetary health a new research area in its own right in 2017, requiring multidisciplinary, interdisciplinary, and transdisciplinary efforts to address unprecedented challenges. This relatively new integrating concept focuses on preserving “the health of human civilization and the state of the natural systems on which it depends” (201) and is gaining support from reputable funding and charitable organizations (e.g., the Rockefeller Foundation). Good human health cannot be preserved when the ecological systems that sustain life on Earth are in a poor or unsatisfactory condition (202). Planetary health is also considered a social movement that aims to transform current practices of living and conducting business at all levels (i.e., individual, societal, national, regional, global), in response to threats to human wellbeing, the sustainability of human civilization, and the health of the planet we inhabit and share with other species (200). Current evidence supports the assertion that global warming is human-induced (203). Religious fasting may help in tackling some environmental issues. For example, during some religious fasting patterns (i.e., Muslim, Buddhism, Daniel), alcoholic drinks are not permitted, and in some Muslim countries (e.g., Tunisia), and are even regulated by a law to not be on sale during the month of Ramadan. It should be acknowledged that some alcoholic beverages (e.g., Whiskey, Vodka) are classified by the NOVA system (204) as ultra-processed foods (UPFS). UPFs include multiple industrial processing (e.g., extrusion, molding, and pre-frying) and manufacturing stages, usage of a large variety of components/additives which have potential dual detrimental impacts on the environment and health (205), and the usage of extensive synthetic packaging, a major source of environmental waste production, mostly containing compounds with carcinogenic and endocrine disruptor properties, UPFs seems to have the most harmful impact, not only on health, but also on the environment (206). It appears that decreasing the consumption of alcoholic drinks during religious fasting is beneficial for environmental health.

It is well-known that livestock is responsible for a significant portion of GHG emissions (14). Marinova and Raphaely (207) found that replacing meat (i.e., beef) with a plant-based option (i.e., wheat) generates 113 times less GHG emissions per nutrient. In addition, red meat is detrimental to planetary health through its direct impacts on land use and freshwater withdrawals (202). Meat consumption is also a significant contributor to biodiversity loss (208), and phosphorus depletion, which endangers future plant-based food production (209). Additionally, based on 800 studies, red meat was classified as carcinogenic to humans (Category 1 for processed meat and Category 2a for cooked meat) by the World Health Organization in 2015 (WHO, 2015). Clearly, red meat consumption is an issue that requires immediate attention in this social movement for restoring and preserving planetary health.

Red meat consumption is not permitted in some religious fasting, including COC fasting, Daniel fast, and Buddhist fasting, which may contribute to the planetary health preservation. However, observers of Ramadan are permitted consume red meat. It is, therefore, necessary to find a strategy that will enable the reduction of its consumption by the Muslim community. In this context, UNEP (210) reported religion continues to play a significant role in many societies including education, health, and politics, and could help to address environmental challenges. Indeed, elements from religion can influence public opinion on sustainability by changing adherents' daily practices, and providing ethical orientation and visions for addressing climate, environmental, and ecological crises. For example, Barclay (211) reported a Muslim fishing community from Tanzania implemented sustainable fishing methods resultant of scientific information provided by Islamic leaders on the subject. Fortunately, “Muslim eco-theologians believe that they have a personal and spiritual responsibility to prevent the spread of environmental degradation because Islam encompasses not only humanity but also nature” (212). In addition, the duty to care for and protect nature is rooted in the Roman Catholic Church's Encyclical “Laudato Si” (213), and Pope Francis has publicly spoken of ecological sins when referring to the climate crisis (214). Likewise, the Interfaith Rainforest Initiative, which fights rainforest deforestation and protects indigenous peoples, uses religious texts from member religions (e.g., Buddhism, Catholicism, Hinduism, Islam, Judaism, Protestant) to advocate for protection (215).

In this context, Weder et al. (216) reported that communication based on religious texts is an effective tool for raising public awareness and shaping public opinion on sustainable development and the environment. Making the public aware of the environmental crisis during prayers is recommended. However, this can be achieved only when religious leaderships (e.g., Imam in the Islamic religion, Pope in the Christian religion) are appropriately educated.

Religious leaders should orient their dialogue toward the obligation to foster efforts of religious believers to decrease pollution, which is responsible for approximately 9 million deaths per year (217). For example, encouraging religious believers to limit (when possible) the use of fuel-powered means of transport will contribute to the decrease of ambient-air pollution, as well as tackling sedentariness. It is worth noting that, in the Muslim religion, avoiding means of transport to join a mosque is encouraged.

Religious leaders should pay attention to food security, which exists only when all individuals have access to physically, socially, and economically sufficient, nutritious, and safe food that meets their dietary needs (218). In this context, Islam does not encourage overeating during and outside the month of Ramadan. Therefore, religious leaders should focus their discussions on avoiding overeating, which could help to ensure food security.

Collectively, religious fasting can help tackle current environmental issues due to the restriction/decrease of the intake of some foods harmful to planetary health. Religious-based communication/dialogue must assert landscape values of respect, love, and care for current and future generations, the planet, and other species. Religiosity and, more broadly speaking, spirituality, are topics, generally, narrowly articulated and framed and have rarely been investigated at the intersection of other topics, such as health, from the individual, public, global, and planetary perspectives. Rethinking religiosity/spirituality means dissecting its profound impacts on human life and daily activities, as well as it means reflecting on public, global, and planetary health from “unusual” points of view. Both religion and health should be conceived and understood, not as abstract entities, but rather as interconnected parts of everyday life, like all those “activities that make us human and keep us in motion: worship, travel, work, migration, the quest for health, and encounters with disease and death” (219). Integrating and incorporating elements of religiosity/spirituality and spiritual, religious, and faith-based practices in public, global, and planetary policies, fully respecting cultural traditions, cultivating and fostering religious and interfaith dialogue, and embracing diversity and pluralism as well as shifting toward more sustainable lifestyles, including dietary intake, may contribute to a more just and equitable society. In conclusion, fasting represents a fundamental “religious health asset,” that is to say, “an asset located in or held by a religious entity that can be leveraged for the purposes of development of public health” (220). Religion-based fasting can be, indeed, mobilized to improve individual health, as well as the health of communities, populations, and the planet.

## Conclusions

Religious fasting is universally widespread—from Buddhism, Jainism, and Hinduism to Christianity, Islam, Judaism, and Taoism—and not the prerogative of a single religious belief. Individual/clinical, public, global, and planetary health have usually been investigated separately and siloed from each other. Religious fasting, alongside other religious health assets, can provide several opportunities across multiple levels of scale, ranging from the individual to the community, population, environmental, and planetary levels, by facilitating and supporting societal transformations and changes, including the adoption of healthier, more equitable, and sustainable lifestyles, preserving the Earth's systems, and addressing major interrelated, cascading and compound challenges, with tremendous impacts on the “whole health”—from inequities and inequalities to NCDs and the climate crisis (221).

## Data availability statement

The original contributions presented in the study are included in the article/Supplementary material, further inquiries can be directed to the corresponding author.

## Author contributions

KT, SG, and NB conceived and designed the manuscript. AA, MB, LP, ES, OB, SK, CC, JG, OA, HJ, and HC critically revised the manuscript. All authors contributed to the article and approved the submitted version.

## Conflict of interest

The authors declare that the research was conducted in the absence of any commercial or financial relationships that could be construed as a potential conflict of interest.

## Publisher's note

All claims expressed in this article are solely those of the authors and do not necessarily represent those of their affiliated organizations, or those of the publisher, the editors and the reviewers. Any product that may be evaluated in this article, or claim that may be made by its manufacturer, is not guaranteed or endorsed by the publisher.
