# Supplementary material for: Religious fasting and its impacts on individual, public, and planetary health: Fasting as a “religious health asset” for a healthier, more equitable, and sustainable society
Source: Front Nutr. 2022 Nov 24;9:1036496. doi: 10.3389/fnut.2022.1036496 (PMC9729557; doi:10.3389/fnut.2022.1036496)
Supplement: Supplementary file 1 [file Data_Sheet_1.docx]

Table S1. Full search strategy for systematic reviews with/without meta-analyses and scoping reviews

|  | **PubMed (28 August 2022)** |  |
| --- | --- | --- |

|  | **Web of Science (28 August 2022)** |  |
| --- | --- | --- |
| **[1]** | religious fasting OR faith-based fasting OR Ramadan OR Ramadan observance OR Ramadan fasting OR Ramadan intermittent fasting OR Islamic fast OR Orthodox fast OR Buddhism OR Jewish OR Hinduism OR Dawood OR Daniel OR Christian Orthodox OR Jainism OR Judaism OR Taoism | **[1] AND [2] AND [3] NOT [4]**  **N= 131** |
|  | N= 88,893 |  |
| **[2]** | health OR mental health OR sleep OR biomarker OR physiology OR immunity OR immune system OR inflammation OR hematology OR food OR nutrition OR adiposity OR body composition OR hydration status OR dehydration OR diabetes OR cardiovascular diseases OR hypertension OR cancer OR chronic kidney disease OR lipid metabolism OR dyslipidemia OR glucose metabolism OR neurodegenerative diseases OR endocrine system OR hormones |  |
|  | N=9,739,475 |  |
| **[3]** | systematic review OR systematic literature review OR meta-Analysis OR systematic review of the literature OR meta‐analytical review OR systematic review and meta-analysis OR scoping review |  |
|  | N= 542,021 |  |
| **[4]** | sport OR athlete OR exercise OR physical activity OR player OR amateur OR recreational OR professional |  |
|  | N= 1,600,811 |  |

|  | **Scopus (28 August 2022)** |  |
| --- | --- | --- |
| **[1]** | “religious fasting” OR “faith-based fasting” OR Ramadan OR “Ramadan observance” OR “Ramadan fasting” OR “Ramadan intermittent fasting” OR “Islamic fast” OR “Orthodox fast” OR Buddhism OR Jewish OR Hinduism OR Dawood OR Daniel OR “Christian Orthodox” OR Jainism OR Judaism OR Taoism | **[1] AND [2] AND [3] NOT [4]**  **N= 171** |
|  | N= 84,512 |  |
| **[2]** | health OR “mental health” OR sleep OR biomarker OR physiology OR immunity OR “immune system” OR inflammation OR hematology OR food OR nutrition OR adiposity OR “body composition” OR “hydration status” OR dehydration OR diabetes OR “cardiovascular diseases” OR hypertension OR cancer OR “chronic kidney disease” OR “lipid metabolism” OR dyslipidemia OR “glucose metabolism” OR “neurodegenerative diseases” OR “endocrine system” OR hormones |  |
|  | N=15,821,101 |  |
| **[3]** | “systematic review” OR “systematic literature review” OR “meta-Analysis” OR “systematic review of the literature” OR “meta‐analytical review” OR “systematic review and meta-analysis” OR “scoping review” |  |
|  | N= 615,254 |  |
| **[4]** | sport OR athlete OR exercise OR “physical activity” OR player OR amateur OR recreational OR professional |  |
|  | N= 2,339,872 |  |

|  | **Cochrane (28 August 2022)** |  |
| --- | --- | --- |
| **[1]** | religious fasting OR faith-based fasting OR Ramadan OR Ramadan observance OR Ramadan fasting OR Ramadan intermittent fasting OR Islamic fast OR Orthodox fast OR Buddhism OR Jewish OR Hinduism OR Dawood OR Daniel OR Christian Orthodox OR Jainism OR Judaism OR Taoism | **[1] AND [2] AND [3] NOT [4]**  **N= 2** |
|  | N= 2 |  |
| **[2]** | health OR mental health OR sleep OR biomarker OR physiology OR immunity OR immune system OR inflammation OR hematology OR food OR nutrition OR adiposity OR body composition OR hydration status OR dehydration OR diabetes OR cardiovascular diseases OR hypertension OR cancer OR chronic kidney disease OR lipid metabolism OR dyslipidemia OR glucose metabolism OR neurodegenerative diseases OR endocrine system OR hormones |  |
|  | N= 6167 |  |
| **[3]** | systematic review OR systematic literature review OR meta-Analysis OR systematic review of the literature OR meta‐analytical review OR systematic review and meta-analysis OR scoping review |  |
|  | N= 4542 |  |
| **[4]** | sport OR athlete OR exercise OR physical activity OR player OR amateur OR recreational OR professional |  |
|  | N= 1528 |  |

|  | **Scielo (28 August 2022)** |  |
| --- | --- | --- |
| **[1]** | religious fasting OR faith-based fasting OR Ramadan OR Ramadan observance OR Ramadan fasting OR Ramadan intermittent fasting OR Islamic fast OR Orthodox fast OR Buddhism OR Jewish OR Hinduism OR Dawood OR Daniel OR Christian Orthodox OR Jainism OR Judaism OR Taoism | **[1] AND [2] AND [3] NOT [4]**  **N= 0** |
|  | N= 5 |  |
| **[2]** | health OR mental health OR sleep OR biomarker OR physiology OR immunity OR immune system OR inflammation OR hematology OR food OR nutrition OR adiposity OR body composition OR hydration status OR dehydration OR diabetes OR cardiovascular diseases OR hypertension OR cancer OR chronic kidney disease OR lipid metabolism OR dyslipidemia OR glucose metabolism OR neurodegenerative diseases OR endocrine system OR hormones |  |
|  | N= 8219 |  |
| **[3]** | systematic review OR systematic literature review OR meta-Analysis OR systematic review of the literature OR meta‐analytical review OR systematic review and meta-analysis OR scoping review |  |
|  | N= 4069 |  |
| **[4]** | sport OR athlete OR exercise OR physical activity OR player OR amateur OR recreational OR professional |  |
|  | N= **120 663** |  |

|  | **EBSCO host (28 August 2022)** |  |
| --- | --- | --- |
| **[1]** | religious fasting OR faith-based fasting OR Ramadan OR Ramadan observance OR Ramadan fasting OR Ramadan intermittent fasting OR Islamic fast OR Orthodox fast OR Buddhism OR Jewish OR Hinduism OR Dawood OR Daniel OR Christian Orthodox OR Jainism OR Judaism OR Taoism | **[1] AND [2] AND [3] NOT [4]**  **N= 1** |
|  | N= 370 |  |
| **[2]** | health OR mental health OR sleep OR biomarker OR physiology OR immunity OR immune system OR inflammation OR hematology OR food OR nutrition OR adiposity OR body composition OR hydration status OR dehydration OR diabetes OR cardiovascular diseases OR hypertension OR cancer OR chronic kidney disease OR lipid metabolism OR dyslipidemia OR glucose metabolism OR neurodegenerative diseases OR endocrine system OR hormones |  |
|  | N= 2371 |  |
| **[3]** | systematic review OR systematic literature review OR meta-Analysis OR systematic review of the literature OR meta‐analytical review OR systematic review and meta-analysis OR scoping review |  |
|  | N= 30 |  |
| **[4]** | sport OR athlete OR exercise OR physical activity OR player OR amateur OR recreational OR professional |  |
|  | N= 1957 |  |

Table S2. Characteristics and main results of the included systematic reviews with/without meta-analyses and scoping reviews

| **Author & year** | **Parameters** | | **Type of review** | **Databases** | **Search period** | **Sample size** | **Age (years)** | **Health status (Included studies)** | **Countries (included studies)** | **Publication date range** | **Quality assessment tool** | **Outcome (included studies, participants)** | **Effect size (95% CI, p value) I^2^ (Chi^2^, p value)/Main results** |
| --- | --- | --- | --- | --- | --- | --- | --- | --- | --- | --- | --- | --- | --- |
| **Kul et al. (2014)** | Anthropometric and metabolic markers | Body mass | SR & MA | MEDLINE, CINAHL, EMBASE | Up to June 2012 | 1476 | 16-76 | Healthy population (n=31) | Iran (n=8); Jordan (n=4); Turkey (n=3); Bahrain (n=2); kuwait (n=2); Pakistan (n=2); Sudan (n=1); Malaysia (n=1); Tunisia (n=1); India (n=1); Morocco (n=1); Egypt (n=1); Bangledesh (n=1); Saudia Arabia (n=1); Indonesia+Japan (n=1) ; Algeria (n=1) | 1978- 2012 | NOS | (21,830) (531 men; 299 women) | Pooled SMD=−0.17 (95%CI −0.26 to −0.07, p=0.001) (I^2^=0 %, p= 0.82)  Men SMD *vs*. Women SMD: NS |
|  |  | TC |  |  |  |  |  |  |  |  |  | (20,806) (581 men; 225 women) | Pooled SMD=−0.29 (95 %CI −0.57 to 0.00, p=0.050) (I^2^=86 %, p= 0.001)  Men SMD *vs*. Women SMD: NS |
|  |  | HDL-C |  |  |  |  |  |  |  |  |  | (13,661) (462 men; 199 women) | Pooled SMD=0.73 (95 %CI −0.10 to 1.57, p=0.09) (I^2^=98 %, p= 0.001) Men SMD *vs*. Women SMD: NS |
|  |  | LDL-C |  |  |  |  |  |  |  |  |  | (13,740) (500 men; 240 women) | Pooled SMD=−1.67 (95 %CI −2.48 to −0.86, p=0.001) (I^2^= 98%, p=0.001)  Men SMD *vs*. Women SMD: NS |
|  |  | TG |  |  |  |  |  |  |  |  |  | (19,828) (538 men; 290 women) | Pooled SMD=−0.20 (95 %CI −0.44 to 0.04, p=0.100) (I^2^=82 %, p= 0.001)  Men SMD *vs*. Women SMD: NS |
|  |  | FG |  |  |  |  |  |  |  |  |  | (16,776) (611 men; 165 women) | Pooled SMD=−1.10 (95 %CI −1.62 to −0.58, p=0.001) (I^2^= 95%, p= 0.001)  Men SMD *vs*. Women SMD: NS |
| **Sadeghirad et al. (2014)** | Anthropometric parameters | Body mass | SR & MA | PubMed/ MEDLINE, CINAHL, EbscoHOST | Between October and November 2011 | 1258 | Range : 18-58 Mean= 29.4 ± 6.4 | .Healthy adults (N=35) | UK (n=2), UK+Sudan (n=1), Malaysia (n=1), Syria (n=2), Israel (n=2), Tunisia (n=3), Morocco (n=2), France (n=1), Kuwait (n=2), Iran (n=3), Bahrain (n=2), Bangladesh (n=1), Turkey (n=4), Saudi Arabia (n=1), Indonesia (n=1), Jordan (n=2), UAE (n=1), Algeria (n=1), NM (n=3) | 1982-2011 | Modified NOS | (34,1234) | Body mass:  Reduction during Ramadan: MD=−1.24 kg (95 %CI −1.60 to −0.88 Kg; p<0.001) (I^2^=79·1 %)  Increase after Ramadan: MD=0.72 kg (95 %CI 0.32 to 1.13 kg; p<0.001) (I^2^=81.7 %)  -After Ramadan *vs*. Baseline: MD=−0.27 kg (95 % CI −0.51, −0.04 kg; p= 0.023) (I^2^=4.1 %)  -Baseline *vs*. During Ramadan:  Men MD=−1.51 Kg (95%CI −2.04 to −0.98 Kg, p<0.001) (I^2^=75%) Women MD=−0.92 kg (95%CI −1.37 to −0.48 Kg, p<0.001) (I^2^= 10.1%)  -Baseline *vs*. After Ramadan:  Men MD= −0.10 kg (95%CI −1.02 to 0.82 Kg, p=0.83) (I^2^=53.5) -Baseline *vs*. After Ramadan:  Women MD= −0.55 (95%CI −1.76 to 0.65 Kg, p=0.37) (I^2^<1%) -During *vs*. After Ramadan:  Men MD=1.02 Kg (95% CI 0.42 to 1.63 Kg, p=0.001) (I^2^= 56.9%) -During *vs*. After Ramadan:  Women MD=−0.04 Kg (95%CI −0.52 to 0.44 Kg, p=0.87) (I^2^<1%) |
| **Bragazzi, (2014)** | Renal function | Renal parameters | SR | WoS, Scopus, PubMed/ MEDLINE, Google Scholar, DOAJ, EbscoHOST, Scirus, ProQuest | NM | 2532 | 26.4 to 54.0 (SD=14.2) | Patients with chronic kidney pathology (n=25) | Saudi Arabia (n=10), Qatar (n=1), Iran (n=6), Tunisia (n=2), Algeria (n=1), Kuwait (n=1), Egypt (n=1), Turkey (n=1), UAE (n=1), Libya (n=1) | 1989- 2013 | NM | Kidney transplant (14,463), renal colic (6,1262), CKD (5,140) | 1) Recipients of kidney allograft can safely fast during Ramadan. 2) Findings are mixed and controversial for patients with nephrolithiasis and CKD.  3) Information about Ramadan fasting falling in hot seasons are scarce. |
| **Bragazzi et al. (2015a)** | Infectious diseases | Infectious diseases biomarkers | SR | WoS, Scopus, MEDLINE/PubMed, Google Scholar, DOAJ, EBSCOhost, Scirus, ProQuest | NM | NM | NM | Patients with infectious diseases (N=51) | NM | 1954-2015 | NM | Antibiotics usage (n=1,34), appendicitis (n=3,4021), diabetes (n=5), diarrhea (n=1), eye infections (n=1,60), Hepatitis (n=2), HIV (n=3), Hookworm and tropical infections (n=1), Meningitis (n=1), Ulcer disease (n=31), Urinary infections (n=2) | 1) Diabetics at risk of developing infectious complications should not fast.  2) Ramadan fasting has little effect on diarrheal patients.  3) For HIV patients, ad hoc drug combinations should be recommended to patients.  4) HIV patients should be advised not consume fatty meals.  4) Ramadan fasting has no effect on the effectiveness of anti-helminthic therapy.  5) Patients with active ulcers should not fast. |
| **Bragazzi et al. (2015b)** | CKD | GFR | SR & MA | Web of Science, Scopus, MEDLINE/PubMed, Google Scholar, DOAJ, EBSCOhost, Scirus, ProQuest | NM | NM | 39.5 ± 13.2 to 54± 14.2 | Patients with CKD (N=6) | Saudi Arabia (n=3), UAE (n=1), Iran (n=1), Egypt (n=1) | 2007-2014 | NM | Cold season (n=4), Hot season (n=2) | SMD= 0.00±0.098 (95%CI −0.19 to 0.19, p=0.99) (I^2^=0.00%)  SMD Hot season *vs*. SMD cold season: NS |
| **Turin et al. (2016)** | CVD events | Congestive heart failure | SR & MA | MEDLINE Embase, EBM Reviews (including Cochrane), PubMed, PubMed Central, Scopus, WoS, CINAHL, Nursing Reference Center, OpenDOAR, health sciences online, turning research into practice, OAIster (WorldCat), Canadian Institute for Health Information, Public Health Agency of Canada, Health Canada, NIH | NM | NM | NM | Patients with Cardiovascular diseases (N=15) | Egypt (n=2), Qatar (n=6), Turkey (n=5), KSA (n=1), Iran (n=1) | 1999-2014 | Quality assessment tool for observational cohort and cross-sectional studies | (2,3179) | During *vs*. before Ramadan:  -Ischemic stroke OR= 0.94 (95%CI 0.71 to 1.26) (I^2^= 4%, p=0.3074)  -Hemorrhagic stroke OR= 1.04 (95%CI 0.76 to 1.41) (I^2^=4.5%, p=0.3062)  During *vs*. other months of the year: -Ischemic stroke OR=0.83 (95%CI 0.60 to 1.16) (I^2^= 0%, p= 0.982)  -Hemorrhagic stroke OR=1.20 (95% CI 0.86 to 1.66) (I^2^= 0%, p= 0.9726) |
|  |  | Acute myocardial infarction |  |  |  |  |  |  |  |  |  | (5,10986) |  |
|  |  | Stroke |  |  |  |  |  |  |  |  |  | (8,12361) |  |
| **Adawi et al. (2017)** | Immune system | Immune system biomarkers | SR | WoS, Scopus, PubMed/MEDLINE, Google Scholar, DOAJ, EbscoHOST, Scirus, Science Direct, Cochrane Library, ProQuest, Google Scholar | NM | 1734 | 15 to 70 | Healthy individuals (N=25) and patients (N=20) | Iran (n= 18), Tunisia (n=6), Turkey (n=4), Indonesia (n=3), Saudi Arabia (n=3), Egypt (n=3), Jordan (n=2), France (n=1), India (n=1), Iraq (n=1), Nigeria (n=1), Qatar (n=1), UAE (n=1) | 2000-2017 | NM | Healthy subjects (N=25), individuals suffering from autoimmune diseases (N=5), patients with cardiac diseases (N=3), pregnant women (n=1) and athletes (N=8), people with HIV/acquired immunodeficiency syndrome (N=1), patients with psychiatric disorders (i.e., schizophrenia) (N=1), asthmatic people (N=1) | 1) Ramadan fasting mildly influences the immune system.  2) The alterations induced are transient (returning to basal pre-Ramadan status shortly afterward).  3) Ramadan fasting during the second trimester of pregnancy is safe with no negative fetal outcomes, or maternal oxidative status alterations. 4) In cardiac patients, Ramadan fasting could improve the lipid profile and alleviate oxidative stress.  5) In asthmatic, HIV and autoimmune disorders patients, fasting during Ramdan is safe.  6) In psychiatric patients (e.g., schizophrenia), fasting could increase immunologic markers. |
| **Koufakis et al. (2017)** | Anthropometric and cardiometabolic parameters. | Lipids profile (i.e., TC, HDL-C and LDL-C levels) | SR | Ovid MEDLINE, PubMed, CINAHL, ProQuest Public Health, Global Health Ovid, Science Direct | NM | 724 | 29.2 ± 1.6 to 43.6 ± 13.2 | Healthy adults (No chronic disease) (N=10) | Greece (n=9), Egypt (n=1) | 2002-2017 | NM | Control group (6,248) Fasters (10,407) | - During OF periods there are:  -Restriction in total energy and fat intake.  -An increase in carbohydrate and fiber consumption.  -For protein intake, results are mixed.  - The overall effect of OF on lipid profile seems to be optimal, with the reduction of TC and LDL-C levels: consistent finding across studies (up to 17.8 and 31.4%, respectively).  - The effect on HDL-C is still unclear.  - The effects of OF on body mass and glucose homeostasis cannot be drawn.  - Any potential negative aspects of OF, attributed to reduced dietary intake of vitamin D and B12 and minerals (mainly calcium), require further studies. |
| **Glazier et al. (2018)** | Pregnancy and perinatal outcomes | Birth weight | SR & MA | EMBASE, MEDLINE, CINAHL, WoS, the Health Management Information Consortium and Applied Social Sciences Index and Abstracts, Google Scholar | On 11 April 2018 | 31374 | NM | Healthy  Pregnant women (n=22) | Iran (n=8), Saudi Arabia (n=1), Lebanon (n=1), Turkey (n=6), UK (n=3), Egypt (n=2), Indonesia (n=1) | 1989- 2018 | ROBINS-I tool | (21,31441) of which 19030 were exposed to Ramadan fasting. | SMD=0.03 (95%CI 0.00 to 0.05) (I^2^= 72.7%, p= 0.0001) |
|  |  | Preterm delivery |  |  |  |  |  |  |  |  |  | (5,5600) of which 1193 were exposed to Ramadan fasting. | OR=0.99 (95% CI 0.72 to 1.37) (I^2^= 0%, p=0.949) |
|  |  | Placental weight |  |  |  |  |  |  |  |  |  | (3,17986) | SMD=−0.94 (95%CI −0.97 to − 0.90) (I^2^= 98.5%, p= 0.0001) |
| **Fernando et al. (2019)** | Body mass Changes | Weight | SR & MA | MEDLINE, PREMEDLINE, EMBASE, Scopus, CINAHL, Global Health. | Up to May 2018 | 2947 | 16-70 | Overweight or obesity and Normal weight (N total=70 articles included) | Sudan (n=1), Malaysia (n=4), Jordan (n=6), Iran (n=11), USA (n=1), Israel (n=1), Morocco (n=1), Tunisia (n=7), UK (n=3), Turkey (n=9), Bahrain (n=2), Bangladesh (n=1), Egypt (n=1), Saudi Arabia (n=3), Indonesia (n=2), Pakistan (n=3), UAE (n=2), Qatar (n=2) Spain (n=2), India (n=2), Australia (n=1), Canada (n=2), Germany (n=1), Thailand (n=1) | 1982-2018 | NM | (63,2759) | -Overall MD=−1.34 kg (95% CI −1.61 to −1.07 Kg, p<0.001) (I^2^ = 40.6%, p<0.001)  -MD normal weight *vs*. MD overweight/obese: NS  -Overall MD (both female and male subgroups) =−1.47 Kg (95% CI −1.82 to −1.12 Kg, p=0.001)  -MD male vs. MD female: NS |
|  | Body Composition Changes | Body fat percentage |  |  |  |  |  |  |  |  |  | (16,709) | -Overall MD=−1.07% (95%CI −1.55 to −0.59, p<0.001) (I^2^=61.5%, p< 0.001);  -MD normal weight *vs*. MD overweight/obese: NS  -Overall MD (both female and male subgroups) =−0.74% (95%CI −1.01 to −0.48 %, p=0.001)  -MD male vs. MD female: NS |
|  |  | Absolute fat mass |  |  |  |  |  |  |  |  |  | (13,640) | -Overall MD=−0.98 Kg (95%CI −1.05 to −0.92 Kg, p=0.001) (I^2^=0.0%, p=0.600).  -Overall MD (both female and male subgroups) =−0.99 Kg (95%CI −1.05 to −0.93 Kg, p < 0.001)  -MD male vs. MD female: NS |
|  |  | Fat-free Mass |  |  |  |  |  |  |  |  |  | (13,572) | -Overall MD=−0.66 kg (95%CI −0.75 to −0.57, p < 0.001) (I^2^= 0.0%, p = 0.753).  -Overall MD (both female and male subgroups) =−0.65 Kg (95%CI −0.75 to −0.54 Kg, p=0.001)  -MD male vs. MD female: NS |
| **Faris et al. (2019)** | Inflammatory markers | IL-1 | SR & MA | PubMed/MEDLINE, WoS ProQuest Medical, Scopus, EBSCOhost, CINAHL, Cochrane, Science Direct, Google Scholar | Up to March 2018 | 311 | 12-70 Median age=38 | Healthy individuals (n=12 studies) | Iran (n=3), Turkey (n=2), Saudi Arabia (n = 2), Jordan (n = 1), UAE (n = 1), Denmark (n = 1), Netherlands (n = 1), Indonesia (n = 1) | 2000-2017 | NM | (2,70) | Hedges' g=−0.016 (95%CI −0.970 to 0.939, p=0.975) (I^2^=0.0%) |
|  |  | IL-6 |  |  |  |  |  |  |  |  |  | (4,110) | Hedges' g=−0.407 (95%CI −0.597 to −0.216, p=0.0001) (I^2^= 0.0%) |
|  |  | TNF-α |  |  |  |  |  |  |  |  |  | (4,102) | Hedges' g=−0.371 (95% CI −0.999 to 0.258, p=0.248) (I^2^=19.7%) |
|  |  | CRP/hs-CRP |  |  |  |  |  |  |  |  |  | (5,111) | Hedges' g=−0.119 (95% CI −0.676 to 0.439, p= 0.677) (I^2^= 26.9%) |
|  | Oxidative stress marker | MDA |  |  |  |  |  |  |  |  |  | (4,117) | Hedges' g=−0.219 (95% CI −0.576 to 0.139, p=0.230) (I^2^=0.0%) |
| **Aydin et al. (2019)** | Glycemic parameters and BMI | BMI | SR & MA | MEDLINE EBSCOhost, EMBASE, Google Scholar | From January 2010 to August 2017 | 2457 | 18-80 | Type 2 diabetic patients (N=19) | Singapore (n=1), USA (n=1), Turkey (n=5), Kuwait (n=1), India (n=1), Pakistan (n=1), UK (n=2), Algeria (n=1), Morocco (n=2), Oman (n=1), Lebanon (n=1), Indonesia (n=1), Malaysia/UK/France/Saudi Arabia (n=1) | 1993-2017 | NOS | (9,683) (210 single OAD, 139 Multi OAD and 334 Multi treatments) | -Single OAD  SMD=−0.00 (95%CI −0.20 to 0.19, p=0.96) (I^2^=0%, p=0.87)  -Multi OAD  SMD=0.11 (95%CI −0.13 to 0.34, p=0.37) (I^2^=0%, p=0.85)  -Multi treatments  SMD=0.03 (95%CI −0.12 to 0.19, p= 0.66) (I^2^ not applicable)  -Pooled SMD=0.04 (95%CI −0.07 to 0.14, p=0.49) (I^2^= 0%, p= 0.98).  -No subgroups differences (p=0.76) |
|  |  | PPG |  |  |  |  |  |  |  |  |  | (10, 199) (108 single OAD and 56 Multi OAD and 35 multi treatments groups) | -Single OAD  SMD=0.01 (95%CI −0.26 to 0.28, p=0.94) (I^2^=0%, p= 0.76) -Multi OAD  SMD=0.00 (95%CI −0.37 to 0.37, p=1.00) (I^2^=0%, p=0.66) -Multi treatments  SMD=0.352 (95%CI −0.15 to 0.80, p= 0.18) (I^2^= 0%, p= 0.69) -Pooled SMD=0.06 (95%CI −0.14 to -0.26, p= 0.54) (I^2^=0%, p= 0.90)  -No subgroups differences (p=0.49) |
|  |  | FPG |  |  |  |  |  |  |  |  |  | (15,624) (364 single OAD, 125 Multi OAD, 135 Multi treatments) | -Single OAD  SMD=0.47 (95%CI 0.20 to 0.74, p<0.001) (I^2^= 52%, p=0.06) -Multi OAD group  SMD=0.36 (95%CI 0.11 to 0.61, p=0.005) (I^2^=0%, p=0.62) -Multi treatments  SMD=0.65 (95%CI 0.03 to 1.27, p=0.04) (I^2^=71%, p= 0.02) -Pooled SMD=0.48 (95%CI 0.27 to 0.70, p<0.001) (I^2^=61%, p=0.001)  -No subgroups differences (p=0.65) |
|  |  | HbA1c |  |  |  |  |  |  |  |  |  | (22, 1173) (884 in single OAD, 254 in multi OAD and 35 in multi treatments) | -Single OAD  SMD=0.03 (95%CI −0.23 to 0.28, p=0.84) (I^2^=84%, p=<0.00001)  -Multi OAD  SMD=0.26 (95%CI 0.03 to 0.49, p=0.03) (I^2^=41%, p=0.10)  -Multi treatments  SMD=0.18 (95%CI −0.29 to 0.65, p=0.45) (I^2^= 0%, p=0.82)  - Pooled SMD=0.13 (95%CI −0.04 to 0.30, p=0.13) (I^2^=71%, p<0.00001)  -No subgroups differences (p= 0.39) |
|  |  | Fructosamine |  |  |  |  |  |  |  |  |  | (15, 998) (943 in single OAD, 39 in Multi OAD and 16 multi treatments) | -Single OAD  SMD=−0.06 (95%CI −0.25 to 0.12, p= 0.49) (I^2^=72%, p<0.00001)  -Multi OAD  SMD=−0.23 (95%CI −0.68 to 0.21, p= 0.30) (I^2^= 0%, p=0.88)  -Multi treatments  SMD=−0.11 (95%CI −0.81 to 0.58, p=0.75) (I^2^ not applicable)  -Pooled SMD=−0.08 (95%CI −0.24 to 0.08, p= 0.32) (I^2^= 65%, p=0.0002)  -No subgroups difference (p=0.78) |
| **Mirmiran et al. (2019)** | Glucometabolic parameters (Lipids and lipoproteins levels) | TG | SR & MA | PubMed, Scopus, and Embase | Up to Feb 2019 | 1327 (37 healthy fasting groups) | 19.25 ± 3.5 to 43.2 ± 9.4 | 33 eligible studies (39 study groups) including 33 apparently healthy groups, 4 pregnant women groups, and 4 studies conducted on athletic subjects | NM | 1978-2019 | JBI Critical Appraisal Checklist for Quasi-Experimental Studies (non-randomized experimental studies) | Healthy (n=29), pregnant (n=2) | -Healthy  MD=0.37 mg/dl (95%CI −5.09 to 5.83 mg/dl) (I^2^= 88, p=0.0001)  -Pregnant  MD=6.31 mg/dl (95%CI −10.19 to 22.81 mg/dl) (I^2^= 0%, p=0.832) |
|  |  | TC |  |  |  |  |  |  |  |  |  | Healthy (n=31), pregnant (n=2) | -Healthy  MD=−1.91 mg/dl (95%CI −6.92 to 3.10 mg/dl) (I^2^=89.3%, p=0.0001)  -Pregnant  MD=7.38 mg/dl (95%CI −3.73 to 18.48 mg/dl) (I^2^= 0%, p=0.581) |
|  |  | HDL-C |  |  |  |  |  |  |  |  |  | Healthy (n=25), pregnant (n=2) | -Healthy MD=−2.41 mg/dl (95% CI −6.57 to 1.76 mg/dl) (I^2^=80.8%, p=0.0001)  -Pregnant  MD=−2.70 mg/dl (95%CI −27.65 to 22.25 mg/dl) (I^2^= 83.4%, p= 0.0001) |
|  |  | LDL-C |  |  |  |  |  |  |  |  |  | Healthy (n=24), pregnant (n=2) | -Healthy  MD=1.81 mg/dl (95%CI 0.55 to 3.07 mg/dl) (I^2^=84.5%, p=0.0001)  -Pregnant  MD=0.08mg/dl (95%CI −3.15 to 3.31) (I^2^=0%, p= 0.663) |
|  |  | VLDL-C |  |  |  |  |  |  |  |  |  | Healthy | -Healthy  MD=−1.56 mg/dl (95%CI=−3.08 to −0.04 mg/dl) |
| **Al mulhem et al. (2020)** | Cardiovascular events | CVD events | SR | EMBASE, MEDLINE, Cochrane library, CINAHL | In April 2018 and updated on April 2019 | 1656 | 63±0. 4 to 64±11.5 | Individuals with diabetes (n=22) CVD events (n=5) CVD risk factors (n=17) | Turkey (n=1), Qatar (n=3), Egypt (n=1) | 1991- 2015 | ROBINS-E | (5,1656) | 1) Insufficient evidence to link Ramadan fasting with increased or reduced incidence of CVD events in diabetics.  2) Stroke risk may increase. |
|  |  | CVD risk factors |  |  |  | 3023 | 43 to 60 |  | Algeria (n=5), Jordan (n=2), Australia (n=1), Egypt (n=1), Qatar (n=2), Pakistan (n=1), Morocco (n=1), NM (n=1), Bangladesh (n=1), Israel (n=1), Mali (n=1), | 1998-2019 |  | (17,3023) |  |
| **Tahapary et al. (2020)** | Glycemic profile | FPG | SR & MA | PubMed/MEDLINE, EBSCOhost, ProQuest | Up to 10 of December 2019 | 27187 | 48.67 to 60.1 | Type 2 diabetes patients (N=28) | Egypt (n=2), Tunisia (n=2), Qatar (n=2), Turkey (n=4), Chad (n=1), Asia/ Europe/Middle East/North Africa (n=1), Algeria (n=4), Singapore (n=3), Saudi Arabia (n=2), UK (n=1), Middle East and North Africa countries (n=1), Middle East/South East Asia (n=1), Indonesia (n=1), Asia and Middle East (n=1), Pakistan (n=1), Iran (n=1) | 1991-2019 | NOS | (11,3446) | MD=−15.28 mg/dl (95%CI −17.22 to −13.34 mg/dl) (I^2^= 79.2%, p<0.001) |
|  |  | HbA1c |  |  |  |  |  |  |  |  |  | (16,7284) | MD=−0.27 mg/dl (95%CI 0.32 to −0.22 mg/dl) (I^2^= 88.0%, p<0.001) |
|  | Lipid profile | TC |  |  |  |  |  |  |  |  |  | (11,3756) | MD=−12.88 mg/dl (95%CI −14.68 to −11.09 mg/dl) (I^2^= 95.54%, p<0.001) |
|  |  | LDL-C |  |  |  |  |  |  |  |  |  |  | MD=−4.42 mg/dl (95%CI −6.17 to −2.66 mg/dl) (I^2^=73.6%, p<0.001) |
|  |  | HDL-C |  |  |  |  |  |  |  |  |  |  | MD=−1.09 mg/dl (95%CI −1.71 to − 0.47 mg/dl) (I^2^= 86.9%, p<0.001) |
|  |  | TG |  |  |  |  |  |  |  |  |  |  | MD=−2.47 mg/dl (95%CI −3.69 to −1.24 mg/dl) (I^2^= 88.3%, p<0.001) |
|  | Anthropometric measurement | Body mass |  |  |  |  |  |  |  |  |  | (15, 2511) | MD=−0.71 kg (95%CI −1.45 to −0.003 Kg) (I^2^= 0%, p= 1.000) |
|  |  | WC |  |  |  |  |  |  |  |  |  | (6,2289) | MD=−0.62 cm (95%CI −1.31 to −0.08 cm) (I^2^= 0%, p=1.000) |
| **Jahrami et al. (2020)** | Body mass changes | Body mass | SR & MA | CINAHL, Cochrane, ProQuest Medical, PubMed/MEDLINE, EBSCOhost, EMBASE, Google Scholar, ScienceDirect, Scopus, WoS | Up to the end of August 2019 | 4176 | 16-80 (median age= 30.0) | Healthy individuals (n=85 studies) | Sudan (n=1), Iran (n=22), Jordan (n=7), Tunisia (n=4), Morocco (n=1), England (n=1), Israel (n=1), Turkey (n=7), Malaysia (n=4), Nigeria (n=2), Saudi Arabia (n=4), Thailand (n=2), UAE (n=4), Pakistan (n=4), Qatar (n=2), India (n=4), Canada (n=2), Iraq (n=1), Spain (n=2), Bangladesh (n=2), Algeria (n=1), Indonesia (n=2), Kuwait (n=2), Bahrain (n=2), Germany (n=1) | 1982-2019 | Standardized checklist | (85,4176) | Hedges' g=−0.360 (95%CI −0.405 to −0.315, p=0.0001) (I^2^=45.6%) MD=−1.022 (95%CI −1.164 to −0.880) |
| **Faris et al. (2020a)** | Metabolic Syndrome Components | WC | SR and MA | CINAHL, PubMed/MEDLINE, Cochrane, ProQuest Medical, EBSCOhost, ScienceDirect, Scopus, and WoS,  Google Scholar, | Up to March 2019 | 4326 | 15–80 (median age 31.5) | Healthy people (N= 85) | Sudan (n=1), Iran (n=28), Saudi Arabia (n=5), Turkey (n=8), Tunisia (n=4), Pakistan (n= 3), Morocco (n=1), Jordan (n=5), UAE (n=2), Algeria (n=1), Kuwait (n=3), Canada (n=2), Malaysia (n=3), Iraq (n=2), Indonesia (n=1), Bangladesh (n=2), Bahrain (n=2), Israel (n=1), India (n=4), Spain (n=2), Belgium (n=1), Nigeria (n=2), Thailand (n=2) | 1982-2019 | Standardized checklist | (24,1557) | Hedge’s g=−0.312 (95%CI −0.387 to −0.236, p=0.0001) (I^2^=49%, p= 0.001) |
|  |  | SBP |  |  |  |  |  |  |  |  |  | (22,1172) | Hedge’s g=−0.239 (95%CI −0.372 to −0.106, p=0.0001) (I^2^=78%, p= 0.001) |
|  |  | FPG |  |  |  |  |  |  |  |  |  | (51,2318) | Hedge’s g=−0.101 (95% CI −0.260 to 0.004, p=0.058) (I^2^=26.6%, p= 0.001) |
|  |  | TG |  |  |  |  |  |  |  |  |  | (63,2862) | Hedge’s g=−0.088 (95% CI −0.171 to −0.004, p= 0.039) (I^2^=78%, p= 0.04) |
|  |  | HDL-C |  |  |  |  |  |  |  |  |  | (57,2771) | Hedge’s g=0.150 (95%CI 0.064 to 0.236, p= 0.001) (I^2^=79%, p=0.001) |
| **Faris et al. (2020b)** | Sleep quality | Daytime sleepiness | SR & MA | Scopus, ScienceDirect, ProQuest Medical, PubMed/MEDLINE, WoS, EBSCOhost, Cochrane, CINAHL, and Google Scholar | Up to the end of June 2019 | 646 | Median age=23.7 | Healthy adults (N=24) | Morocco (n=1), KSA (n=8), UAE (n=1), Algeria (n=1), Turkey (n=1), Israel (n=1), France (n=1), Tunisia (n=4), Qatar (n=3), Singapore (n=1), India (n=1), Germany (n=1) | 2001- 2019 | Standardized checklist | (9,362) | Hedges' g=−0.06 (95%CI −0.43 to 0.28, p= 0.733) (I^2^ = 76%, p= 0.001). |
|  |  | Sleep duration |  |  |  |  |  |  |  |  |  | (22,571) | Hedges' g=−0.43 (95%CI −0.64 to −0.22, p= 0.0001) (I^2^= 78%, p< 0.001) |
| **Faris et al. (2020c)** | Glucometabolic markers | FG | SR & MA | Cochrane, PubMed/MEDLINE, ScienceDirect, CINAHL, ProQuest Medical, EMBASE, EBSCOhost, Scopus, WoS, Google Scholar | Up to January 2020. | 3134 | 16–80 | Healthy population (N=71) | Iran (n=18), Tunisia (n=4), Morocco (n=2), Turkey (n=9), Saudi Arabia (n=8), Algeria (n=1), Iraq (n=1), Canada (n=1), Malaysia (n=2), Jordan (n=4), Pakistan (n=5), Thailand (n=2), India (n=1), Sudan (n=1), UAE (n=3), Belgium (n=1), Nigeria (n=1), Germany (n=1), Israel (n=1), Kuwait (n=2), Bahrain (n=2), Bangladesh (n=1) | 1982-2020 | Standardized checklist | (61,2743) | Hedges' g=−0.102 (95%CI −0.194 to −0.01, p=0.030)  MD=−1.294 mg/dl (95%CI −2.147 to −0.441 mg/dl) (I^2^=81.79%) |
|  |  | Serum insulin |  |  |  |  |  |  |  |  |  | (16,648) | Hedges'g =0.030 (95%CI −0.165 to 0.226, p=0.761)  MD = 0.331 μU/ml (95% CI −2.717 to 3.379 μU/ml, I^2^=82.73%) |
|  |  | Insulin resistance (HOMA-IR) |  |  |  |  |  |  |  |  |  | (10,349) | Hedges' g=−0.012 (95%CI −0.274 to  0.250, p=0.930)  MD=−0.028 (95% CI −0.396 to 0.339, I^2^=83.88%) |
|  |  | leptin |  |  |  |  |  |  |  |  |  | (13,442) | Hedges' g=−0.010 (95%CI −0.243 to 0.223, p=0.932)  MD=0.084 ng/ml (95%CI -0.266 to 0.433 ng/ml, I^2^=83.30%) |
|  |  | Sdiponectin |  |  |  |  |  |  |  |  |  | (11,511) | Hedges' g=0.034 (95% CI −0.227 to 0.296, p=0.798)  MD = 0.096 ng/ml (95% CI −0.686 to 0.494 ng/ml, I^2^=87.78%) |
| **Jahrami et al. (2021)** | Cardiometabolic parameters | TC | SR & MA | EBSCOhost, CINAHL, Cochrane, EMBASE, PubMed/MEDLINE, Scopus, Google Scholar, ProQuest Medical, ScienceDirect, and Web of Science | Up to November 2020 | 4431 | 18-85 (median age= 32.4) | Healthy adults (N=91) | England and Sudan (n=1), Tunisia (n=3), Turkey (n=9), Morocco (n=1), Israel (n=1), Jordan (n=7), Iran (n= 24), Kuwait (n=3), Bahrain (n=2), Bangladesh (n=3), India (n=8), Iraq (n=4), UAE (n=5), Algeria (n=1), Canada (n=2), Pakistan (n=5), Thailand (n=2), Nigeria (n=1), Indonesia (n=2), Saudi Arabia (n=2), Malaysia (n=2), Germany (n=1), NM (n=2) | 1982-2020 | Standardized checklist | (77,3705) | Hedge's g=−0.092 (95%CI −0.168 to 0.016, p=0.017) (I^2^= 80.5%, p=0.001) |
|  |  | TG |  |  |  |  |  |  |  |  |  | (74,3591) | Hedge's g=−0.127 (95%CI −0.203 to 0.051, p=0.001) (I^2^=78%, p=0.001) |
|  |  | HDL-C |  |  |  |  |  |  |  |  |  | (68,3528) | Hedge's g=0.138 (95%CI 0.051 to 0.224, p= 0.002) (I^2^=83.3%, p=0.001) |
|  |  | LDL-C |  |  |  |  |  |  |  |  |  | (65,3354) | Hedge's g=−0.115 (95%CI −0.197 to −0.034, p= 0.006) (I^2^= 80.1%, p= 0.001) |
|  |  | VLDL-C |  |  |  |  |  |  |  |  |  | (13,648) | Hedge's g=−0.252 (95%CI −0.431 to 0.073, p= 0.006) (I^2^= 80%, p= 0.001) |
|  |  | DBP |  |  |  |  |  |  |  |  |  | (32, 1716) | Hedge's g=−0.255 (95%CI −0.363 to 0.147, p= 0.0001) (I^2^=74%, p=0.001) |
|  |  | HR |  |  |  |  |  |  |  |  |  | (12, 674) | Hedge's g=−0.082 (95%CI −0.300 to 0.136, p= 0.460) (I^2^= 85%, p=0.001) |
| **Faris et al. (2021)** | Liver function | AST | SR & MA | Cochrane, CINAHL, EMBASE, EBSCOhost, Google Scholar, PubMed/MEDLINE, ProQuest Medical, Scopus, ScienceDirect, WoS | to the end of July 2020 | 601 | 18-57 | Healthy adults (n=20) | Jordan (n=1), Iran (n=3), Turkey (n=5), UAE (n=1), Iraq (n=3), Saudi Arabia (n=3), Belguim (n=1), India (n=1), Indonesia (n=1), Tunisia (n=1) | 1987- 2020 | Standardized checklist | (16, 502) | SMD=−0.257 (95%CI −0.381 to −0.133, p= 0.0001) (I^2^=42%) |
|  |  | ALT |  |  |  |  |  |  |  |  |  | (16, 502) | SMD=−0.105 (95% −0.282 to 0.07, p= 0.244) (I^2^=71%) |
|  |  | GGT |  |  |  |  |  |  |  |  |  | (2, 46) | SMD=−0.533 (95%CI 0.842 to −0.224, p= 0.001) (I^2^ = 0%) |
|  |  | ALP |  |  |  |  |  |  |  |  |  | (10, 312) | SMD=−0.318 (95%CI 0.432 to −0.204, p= 0.0001) (I^2^=0.0%) |
|  |  | BLU |  |  |  |  |  |  |  |  |  | (10, 325) | SMD=−0.264 (95% −0.520 to −0.007, p= 0.044) (I^2^=70.1%) |
|  |  | LDH |  |  |  |  |  |  |  |  |  | (5,145) | SMD=−0.041 (95%CI −0.380 to 0.298, p= 0.814) (I^2^=72%) |
|  |  | PT |  |  |  |  |  |  |  |  |  | (2,74) | SMD=−0.027 (95% −0.732 to 0.678, p= 0.940) (I^2^=87%) |
| **Gaeini et al. (2021)** | Hormones regulating appetite and satiety | Leptin levels | SR & MA | PubMed, Scopus, Google Scholar, Embase | Up to 2020 | NM | NM | Healthy subjects (n=7), overweight or obese individuals (n=4)  cardiac patients (n=1), pregnant women (n=2), subjects at high risk for diabetes (n=1)  (N=16 in the SR and 10 in the MA) | Tunisia (n=2), Turkey (n=1), Bahrain (n=2), Saudi Arabia (n=2), UAE (n=1), France (n=1), Iran (n=2), Malaysia (n=1), Qatar (n=1), Pakistan (n=1), Gambia (n=1), Germany (n=1) | 2003-2020 | JBI  Critical Appraisal Checklist for Quasi-Experimental Studies | (13,NM) Males (n=5), females (n=6), overweight/obese (n=6) normal weight (n=4) | - Pooled MD=−2.28 ng/ml (95%CI −3.72 to −0.84 ng/ml) (I^2^=94.5%, p=0.0001)  -Normal-weight subjects  WMD=−4.67 ng/ml (95%CI −6.03 to −3.31 ng/ml, p<0.001) (I^2^ =91.2%)  -Overweight/obese subjects  MD=−3.43 ng/ml (95%CI −5.69 to −1.17 ng/ml, p= 0.003) (I^2^=92.4%)  -Men  MD=−1.63 ng/ml (95%CI −3.67 to 0.41 ng/ml, p= 0.118) (I^2^= 69.7%)  -Women  MD=−1.71 ng/ml (95%CI=−3.74 to 0.31 ng/ml, p=0.098) (I^2^ = 96.2%). |
|  |  | Adiponectin levels |  |  |  |  |  |  |  |  |  | (8, 348) | MD=2.19 ng/ml (95%CI −0.29 to 4.67 ng/ml) (I^2^=95.1%) |
| **Berthelot. (2021)** | Psychiatric parameters | Stress | SR & MA | PubMed interface (Medline database), ScienceDirect and Google Scholar. | last search was carried out on 30 August 2021 | 1436 | NM | Individuals without psychiatric disorders (N=11) | Iran (n=2), Turkey (n=1), Kuwait (n=1), Germany (n=3), Malaysia (n=2), Czech Republic (n=1), USA (n=1) | 2011-2019 | Study quality assessment tool for observational cohort or cross-sectional studies for Ramadan studies | (2,386) | -Ramadan studies  SMD=−0.222 (95%CI −0.323 to −0.121, p= 0.0001) (I^2^= 0%) |
|  |  | Anxiety |  |  |  |  |  |  |  |  |  | (4,546) | -Ramadan Studies  SMD=−0.367 (95%CI -0.689 to −0.084, p=0.012) (I^2^=87.79%) |
|  |  | Depression |  |  |  |  |  |  |  |  |  | (5,1009) | Ramadan Studies  SMD=−0.618 (95%CI −0.977 to −0.258, p= 0.001) (I^2^=95.32%) |
| **Kokkinopoulou and Kafatos, 2021** | Dietary patterns | Metabolic syndrome risk factors (blood pressure, blood lipids and anthropometric measurements) | Scoping review | PubMed (via MEDLINE), CINAHL, Scopus, Google Scholar | From February until March 2020. An additional search was performed in June 2020 | 1226 (Fasting participants) | 18- 84 one article focusing on a paediatric population (5-15.5) | Healthy adults (no chronic illnesses) (N=20) | USA (n =2), Egypt (n=2), Greece (n=16) | 2002- 2020 | Checklist of Hawker and colleagues | (20,1226) Faster, control (9,569) | COC fasting resulted in:  - No deficiency in essential amino acid intake.  - Healthier blood lipid profile.  - Decrease in systolic blood pressure, body mass and BMI.  - No deficiencies in macro- and micronutrients intakes. |
| **Al Jafar et al. (2021)** | Blood pressure | SBP | SR & MA | PubMed, EMBASE, Scopus | Up to March 3, 2020. | 3213 | 21.7±0.7 to 66.8±10.3 | - Healthy individuals - Patients with type 2 diabetes  - Patients with hypertension  - CKD patients (N= 33 including LORANS) | Iran (n=7) , Pakistan (n=3), Turkey (n=3), Thailand (n=1), Indonesia (n=1), Jordan (n=1), UAE (n=2), Tunisia (n=1), Spain (n=1), Canada (n=1), Bengal (n=1), India (n=1), NM (n=3), Singapore (n=1), Qatar (n=1), Mali (n=1), Saudi Arabia (n=2), Malaysia (n=1), UK (n=1) | 1996-2019 | NOS | -Healthy individuals (15,749)  -Hypertensive individuals (5,114)  -Diabetics (10,1783)  -CKD patients (7,614) | -Healthy individuals  MD=−3.21 (95%CI −4.79 to −1.64) (I^2^=44%, p= 0.03)  -Hypertensive individuals  MD=−8.44 (95%CI −15.16 to −1.72) (I^2^=0%, p= 0.68)  -Diabetics  MD=−3.53 (95%CI −6.14 to −0.93) (I^2^=61%, p<0.01)  -CKD patients  MD=0.09 (95%CI −2.42 to 2.61) (I^2^= 0%, p= 0.73)  -Pooled results  MD=−3.19 (95%CI −4.43 to −1.96, p<0.01) (I^2^= 48%, p<0.01) |
|  |  | DBP |  |  |  |  |  |  |  |  |  | -Healthy individuals (15,747)  -Hypertensive individuals (5 ,69)  -Diabetics (10 ,1783)  -CKD individuals (7 ,614) | -Healthy individuals  MD=2.82 (95%CI −4.34 to −1.30) (I^2^= 70%, p<0.01)  -Hypertensive individuals  MD=−4.52 (95%CI −7.75 to −1.28) (I^2^= 24%, p= 0.26)  -Diabetics  MD=−2.13 (95%CI −3.59 to −0.67) (I^2^= 61%, p<0.01)  -CKD patients  MD= 0.27 (95%CI −1.19 to 1.73) (I^2^= 1%, p=0.41)  -Pooled results  MD=−2.26 (95%CI −3.19 to −1.34, p<0.01) (I^2^= 66%, p<0.01) |
| **Besbes et al. (2022)** | SFR, inflammatory and metabolic parameters | SFR | SR | PubMed and Scopus | Up to 15 July 2021 | 229 | 24.2 ± 2.3 to 59 | Healthy adults (N=6) | Saudi Arabia (n=1), Turkey (n=1), United Arab Emirates (n= 1), Iran (n=3) | 2004-2020 | JBI Critical Appraisal Tool | (1,0) | -SFR decreased by 10% during Ramadan in fasting subjects. |
|  |  | Melatonin |  |  |  |  |  |  |  |  |  | (1,8) | -The circadian pattern of melatonin did not change during Ramadan.  -Melatonin levels dropped significantly *vs*. baseline. |
|  |  | Cortisol |  |  |  |  |  |  |  |  |  | (2,132) | -The salivary cortisol levels were unchanged or increased during Ramadan. |
|  |  | Glucose |  |  |  |  |  |  |  |  |  | (1, 60) | -The salivary glucose levels decreased. |
|  |  | IgA |  |  |  |  |  |  |  |  |  | (1, 24) | Salivary IgA decreased during the last week of Ramadan. |
|  |  | Uric acid |  |  |  |  |  |  |  |  |  | (1, 35) | -Uric acid decreased during Ramadan. |
|  |  | ALP |  |  |  |  |  |  |  |  |  | (1, 35) | ALP increased during Ramadan. |
|  |  | AST |  |  |  |  |  |  |  |  |  | (1, 35) | AST decreased during Ramadan. |
| **Mousavi et al. (2022)** | Intestinal microbiome | Changes in the gut microbiota | SR | PubMed, Scopus, WoS, Google Scholar | Up to December 2021 | 457 | 18-65 | Healthy, obese and adults with metabolic syndrome | NM | 2015-2021 | NM | (12,457) | -Significant shift in the gut microbiota:  - An increase in the abundance of *Lactobacillus* and *Bifidobacteria* following fasting diets.  - an increase in the bacterial diversity.  -A decrease in inflammation and increased production of some metabolites (e.g., SCFAs) in individuals or samples under fasting diets.  - Ramadan fasting improves health parameters through positive effects on some bacterial strains (e.g., *Akkermansia muciniphila,* *Bacteroide*). |

Abbreviations: ALP: Alkaline phosphatase, ALT: Alanine transaminase, AST: Aspartate transaminase, BLU: Bilirubin, BMI: Body mass index, CKD: chronic kidney disease, CRP: C-reactive protein, CVD: Cardiovascular disease, DBP: Diastolic blood pressure, DBP: diastolic blood pressure, DOAJ: Directory of Open Access Journals, ESS: Epworth sleepiness scale, FG: Fasting Glucose, FPG: Fasting plasma glucose, GFR: Glomerular filtration rate, GGT: Gamma - glutamyl transferase, HbA1c: Glycated hemoglobin, HDL-C: High-density lipoprotein cholesterol, HIV: Human immunodeficiency virus, HR: Heart rate, Hs-CRP: High sensitivity C-reactive protein, IGA: Immunoglobulin A, IL-1: Interleukin-1, IL-6: Interleukin-6, JBI: Joanna Briggs Institute, LDH: Lactate dehydrogenase, LDL-C: Low-density lipoprotein cholesterol, LORANS: London Ramadan study, MA: Meta-analysis, MDA: Malondialdehyde, MD: Mean difference, NOS: New Castle Ottawa scale, NIH: National Institute of Health, NM: Not mentioned, NOS: New Castle Ottawa scale, NS: Not significant, OAD: Oral antidiabetics, OF: Orthodox Fasting, OR: Odds ratios, PPG: Post prandial plasma glucose, PT: Prothrombin time, SBP: Systolic blood pressure, SBP: Systolic blood pressure, SCFAs: Short-chain fatty acids, SD: Standard deviation, SFR: Salivary flowrate, SMD: Standardized mean difference, SR: Systematic review, SR & MA: Systematic review and meta-analysis, TC: Total cholesterol, TG: Triglycerides, TNF: Tumor necrosis factor, UAE: United Arab Emirates, UK: United Kingdom, USA: United states of America, VLDL-C: Very low-density lipoprotein cholesterol, WC: waist Circumference, WoS: Web of Science.

Note: - Hedges’ g value was used to measure effect size (ES): 0.8 reflects a large effect, 0.5 reflects a medium effect, and ≤0.2 was considered a small effect (Hedges, 1981).

- SMD value was used to measure ES: ES < 0.2 reflects a trivial effect, 0.2–0.6 reflects a small effect, 0.6–1.2 reflects a moderate effect, 1.2–2.0 reflects a large effect, ES > 2.0 reflects a very large effect, and ES > 4.0 reflects extremely large effect (Cohen, 1988).
